# Differential Dynamic Changes of Reduced Trait Model for Analyzing the Plastic Response to Drought Phases: A Case Study in Spring Wheat

**DOI:** 10.3389/fpls.2019.00504

**Published:** 2019-04-26

**Authors:** Marwa N. M. E. Sanad, Andrei Smertenko, Kimberley A. Garland-Campbell

**Affiliations:** ^1^Department of Genetics and Cytology, National Research Centre, Giza, Egypt; ^2^Department of Crop and Soil Sciences, Washington State University, Pullman, WA, United States; ^3^Institute of Biological Chemistry, Washington State University, Pullman, WA, United States; ^4^USDA-ARS Wheat Health, Genetics, and Quality Research Unit, Washington State University, Pullman, WA, United States

**Keywords:** ROS homeostasis, dynamic changes, heritability, peroxisome proliferation, spring wheat, drought tolerance, adaptive mechanisms

## Abstract

Current limited water availability due to climate changes results in severe drought stress and desiccation in plants. Phenotyping drought tolerance remains challenging. In particular, our knowledge about the discriminating power of traits for capturing a plastic phenotype in high-throughput settings is scant. The study is designed to investigate the differential performance and broad-sense heritability of a battery set of morphological, physiological, and cellular traits to understand the adaptive phenotypic response to drought in spring wheat during the tillering stage. The potential of peroxisome abundance to predict the adaptive response under severe drought was assessed using a high-throughput technique for peroxisome quantification in plants. The research dissected the dynamic changes of some phenological traits during three successive phases of drought using two contrasting genotypes of adaptability to drought. The research demonstrates 5 main findings: (1) a reduction of the overall dimension of the phenological traits for robust phenotyping of the adaptive performance under drought; (2) the abundance of peroxisomes in response to drought correlate negatively with grain yield; (3) the efficiency of ROS homeostasis through peroxisome proliferation which seems to be genetically programmed; and (4) the dynamics of ROS homeostasis seems to be timing dependent mechanism, the tolerant genotype response is earlier than the susceptible genotype. This work will contribute to the identification of robust plastic phenotypic tools and the understanding of the mechanisms for adaptive behavior under drought conditions.

**Summary statement:**

This study presents the estimated broad-sense heritability of 24 phenological traits under drought compared with non-stressed conditions. The results demonstrated a reduced model of the overall dimension of the phenological traits for phenotyping drought tolerant response including a novel trait (peroxisome abundance). Also, it displays that the adaptive mechanism through peroxisomes proliferation that is a genetic-dependent manner and related to the stress phase, since tolerant plants can sense the stress and maintain the cellular balance earlier than the sensitive plants.

## Introduction

Limited water availability threatens global food security and agricultural productivity. Currently, 28% of the world's arable land is subject to continuous water deficit with an additional 50% experiencing periodic water shortages (Salekdeh et al., 2009; Cramer et al., 2011). Forecasting the global distribution of aridity between 2030 and 2039 uncovered that severe drought is predicted to occur in most of Africa, Southern Europe, Southeast Asia, the Middle East, and North America based on the Palmer Drought Severity Index (Figure S1) (Dai, 2011). Thus, there is a need to enrich the plant phenomics with comprehensive information about the robustness of various components of traits under severe drought and to predict the phenotypic plant response under severe and prolonged drought during the early growth stage.

Plants survive in a range of soil water contents but most optimally near field capacity. A decrease below the minimum balance level limits to water availability to plants, resulting in drought stress (Figure S2) (Werner, 2000). Prolonged drought can cause further reduction of soil moisture to the permanent wilting point (PWP) when the moisture content becomes insignificant, causing desiccation stress (Furr and Reeve, 1945; Taiz and Zeiger, 1991). Drought and desiccation-tolerance in plants require that plants sustain mechanisms to overcome the effect of the extreme water loss on cells for prolonged periods (Berjak, 2006). Adaptive plants under prolonged drought promote stress tolerance in a complex process (Bewley, 1979) which (1) constrains the damage to the cellular components at a repairable level; (2) maintains cellular integrity during stress; (3) drives the repair mechanisms upon hydration.

Drought stress can be assessed using various traits; such as relative water content (RWC) and chlorophyll fluorescence, grain yield, plant biomass and plant height (Rampino et al., 2006; Nouri et al., 2011; Fischer et al., 2012; Munjal and Singh Dhanda, 2016). In plants, overall water loss below 70% of RWC may stimulate the production of reactive molecules and free radicals known as Reactive Oxygen Species (ROS) (Smirnoff, 1993). A correlation between the water deficiency and the stimulation of ROS metabolism has been found in *Lotus japonicus* (Signorelli et al., 2013), and in *Arabidopsis thaliana* (Noctor et al., 2014). Conceivably, ROS homeostasis can be used to assess the cellular response to drought. However, our ability to accurately measure the activity of the ROS-scavenging system remains limited due to the chemical complexity and temporal hierarchy of redox reactions. For example, the excess of ROS production under mild water loss is quenched by enzymes and metabolites of the ascorbate-glutathione cycle (Gamble and Burke, 1984; Noctor and Foyer, 1998) or by certain sugars (Keunen et al., 2013). Quenching the intense flux of ROS requires the elevated activity of scavenging enzymes including ascorbate peroxidase, superoxide dismutase (SOD), and catalase (CAT) (Jiang and Zhang, 2002; Rubio et al., 2002; Guo et al., 2006; Møller et al., 2007). ROS-scavengers are located in all cellular compartments including the cell wall, membranes, cytoplasm, chloroplast, mitochondria, peroxisomes, and the apoplast; however, the oxidative stress mechanism mediated by ROS that stimulates peroxisome proliferation (Palma et al., 1991), and the bulk of ROS metabolism takes place in peroxisomes (Foyer and Noctor, 2003). For this reason, peroxisomes are indispensable for stress tolerance such as the activity of CAT enzyme in wheat (Luna et al., 2004), maize (Jiang and Zhang, 2002), and rice (Guo et al., 2006).

Conversely, CAT deficiency results in the accumulation of ROS and higher susceptibility to high-light stress in tobacco leaves (Willekens et al., 1997). The importance of peroxisomes in ROS neutralization during a stress response suggests that the number of peroxisomes in cells should increase under stress conditions (Smertenko, 2017). In agreement with this hypothesis, many stresses, including high light intensity (Ferreira et al., 1989), ozone (Morre et al., 1990; Oksanen et al., 2004), metal stress (Romero-Puertas et al., 1999), and salt stress (Mitsuya et al., 2010) promote proliferation of peroxisomes and cause higher activity of ROS-catabolizing enzymes. Exogenously applied H_2_O_2_ (Lopez-Huertas et al., 2000; Fahy et al., 2017) as well as stress-induced ROS (Oksanen et al., 2004) trigger the proliferation of peroxisomes. Hence, peroxisome abundance can be used as a proxy of intracellular ROS content. Investigating this theory wasn't be possible without a technique for quantifying peroxisome abundance in plant extracts as our technology (Fahy et al., 2017) using small fluorescent dye Nitro-BODIPY (N-BODIPY) labels plant peroxisomes *in vivo* (Landrum et al., 2010). However, it was important to advance that technique to be utilized in a high-throughput format for the applications of screening plant phenotype in large populations, which was one of the basic objectives in this research. In addition, no systematic analysis of peroxisome proliferation during drought or correlation between peroxisome abundance and other traits is currently available. Here, this research addresses this gap for phenotyping a population of 16 wheat genotypes from geographic locations with distinct precipitation patterns and analyzed the abundance of peroxisomes in response to severe drought. Subsequently, the correlation between peroxisome abundance and 23 traits that are commonly used for phenotyping drought response was analyzed. Such a comprehensive analysis would help significantly to understand the similar phenotypic patterns in order to reduce the phenotypic dimension to the minimum robust traits for phenotyping drought-tolerance.

In a previous study, Cruz de Carvalho (2008) proposed a model to describe the correlation between the levels of the ROS and the antioxidants through three successive phases of drought: (1) the normal levels at the beginning of drought, (2) the drought induction that stimulated the ROS and antioxidants and defense response, and (3) the prolonged drought to include both of the ROS scavenging and programmed cell death (PCD) mechanisms. In barley, the maximum stress considered as the adaptation phase (Ribaut, 2006). Thus, this study uses/adopts that hypothesized model and applies it during a time-course of 14 days of water deficiency using the most and least adaptive genotypes to drought out of the screened population. A set of important phenotypic traits (stomatal conductance, H_2_O_2_ content, root length, and RWC) was monitored and associated with peroxisome abundance to understand the dynamics of these traits during drought phases and their role in drought tolerance mechanisms. The phenotypic assessment is done at the maximum stress and with respect to the programmed developmental plasticity (Sanad et al., 2016). During drought progression, the tolerant and susceptible performances that are associated with the genetic regulation are distinguished. Subsequently, the homologs of peroxisome biogenesis machinery in *T. aestivum* are predicted in order to design primers for investigating the regulation of the gene machinery during drought phases to validate the quantification of peroxisome abundance. The predicted genes are peroxisomal factor11genes (PEX11s), dynamin-related proteins (DRPs), and Mitochondrial fission 1A (FIS1A).

Ultimately, this work aims to present a comprehensive study at an early growth stage to uncover the most robust phenotypic traits that promote the tolerance mechanisms for phenotyping plant plasticity. To this end, the study considers several objectives: (1) to advance the technology of peroxisome proliferation to be applied in high-throughput format, (2) to assess a comprehensive set of traits including peroxisome abundance for phenotyping plant response to drought, (3) to investigate the correlation among the phenological traits, (4) to test the robustness and sensitivity of the phenological traits and the suitability of peroxisome abundance to be considered as a phenotypic trait, and (5) to study the dynamics of the phenological traits and gene regulation of peroxisome proliferation machinery during three successive phases of drought.

## Materials and Methods

### Plant Material and Growth Conditions

A worldwide collection of 16 genotypes of spring wheat was used for assessing the phenological traits under severe drought (*Triticum durum* and *T. aestivum*) (Table S1). All plants were grown in D60H Deepots containers (983 ml; Stuewe and Sons, Oregon, USA), filled with an equal weight of Sunshine Mix#4 potting soil (Sungro, Canada) containing Osmocote 14-14-14 slow release fertilizer (Scotts Co. LLC). Ten day-old-seedlings were individually transferred to pots and grown on a diurnal cycle of 16 light/8 dark h, at 700–1,000 μmol PAR light (1000 W HPS), 22–23°C/16°C, 404 pm CO_2_, 16–17% humidity. The tillering stage was chosen for screening as the most plastic developmental stage (Sanad et al., 2016). The drought stress was applied by withholding watering at the beginning of the tillering stage (Zadoks growth stage 21) (Zadoks et al., 1974). The soil moisture content parameters were measured every 3 days using Decagon soil probe 5TE (Decagon Devices, Inc., Pullman, WA, USA) to measure the (1) the raw volumetric water content (VWC) values (m^3^/m^−3^) which were multiplied by 100 to get the percentage values; (2) the soil temperature (°C); and (3) the bulk electrical conductivity (ds^−1^/m^−1^). No custom calibration was used whereas the mineral soil calibration was used for the potting mix. Out of the population, Onas and Alpowa genotypes were selected to investigate the dynamics of some phenotypic parameters during a time-course of 14 days of progression water deficiency. The same growth conditions and the experimental procedure mentioned above were duplicated.

### Phenotyping Stress Response and Recovery

#### Experiments Workflow

An amount of 21 phenological soil parameters were collected and the survival rate, RWC, PSII, leaf temperature, chlorophyll content, and peroxisome abundance were measured at the maximum stress. The recovery rate and days to heading were recorded. At harvest, morphological traits (Plant height, peduncle length, awn length, root length, and root dry weight), plant biomass (standing biomass and total plant dry weight), and plant yield (number of tillers, the total spike number, spike weight, number of productive tillers, grain number, and grain weight) were measured (Figure S3).

Another experiment was designed to investigating the dynamics of six phenological traits across the time-course of 14 days to cover three phases of drought. Soil moisture, peroxisome content, root length, relative water content, and stomatal conductance were collected up till day 6 of the experiment, then on the 8, 11, and 14th days, respectively. The H_2_O_2_ was measured on the 3rd, 6th, 8th, 11th, and 14th days to represent the ROS content. Three selected time points (3rd, 8th, and 14th days) were selected for quantifying gene expression of peroxisome biogenesis genes. To avoid collecting data in response to plant injury, not only water deficiency, each group of treatment was duplicated 14 times at each of the previously mentioned time points. After the measurements for each day group, the roots were harvested to measure the root length of the main root.

#### Plant Phenotyping Description

Stress was extended until the 25–32 developmental stages of Zadok's scale across the genotypes. The protocol for measuring the phenotypic parameters was followed according to the CIMMYT physiological breeding guidelines (Pask et al., 2012). The RWC was measured at the maximum stress (Stocker, 1929). The measurements of chlorophyll content and photosystem II (PSII) activities were performed on the most recent fully expanded leaf. PSII activity (YII) was calculated using equation YII = F_m_-F_o_/F_m_, where F_m_ is the ground fluorescence of light adapted samples. F_o_ is the maximal fluorescence of light adapted samples, and YII is the yield of photochemical energy conversion (Maxwell and Johnson, 2000). The chlorophyll content was calculated using CCM-200 Plus (OPTI-Sciences, Hudson, NH, USA). While, stomatal conductance was measured using High Precision Leaf Promoter SC-1 (Decagon Devices, Inc., Pullman, WA, USA). Moreover, the survival rate was scored (from 1 to 10) while the recovery rate (from 1 to 10) was scored 12 days after the stress ended. The description of both rates is illustrated here (Figures S4A,B). Later, the number of days to heading was counted from sowing to heading stage (Zadok's growth stage 58). After harvesting, roots were washed and scanned. The total root length was measured using Assess 2.0 image analysis software (Lamari, 2008). Then the roots were dried in the oven at 75°C for 3 days and total dry weight was measured. All parts of the plant, excluding roots, were harvested, dried, and then weighed to measure the relative plant dry weight. While, the standing biomass was calculated in relation to the planting area (Lonsdale, 1990; Proulx et al., 2015).

The content of hydrogen peroxide (H_2_O_2_) represented the ROS content. A 150 mg of leaf samples of Alpowa and Onas genotypes for each day/biological replicate were homogenized under liquid nitrogen conditions. The extraction procedure and the H_2_O_2_ assay was conducted according to the recommended procedures of Junglee et al. (2014). The extracts were placed in a 96 well-UV-microplate and measured at different wavelengths (280, 320, 350, and 390 nm). The measurements were optimized and calibrated at 280 nm, which was the highest and constant peak. The spectrofluorimeter Synergy Neo B plate reader (Biotek Instrument, Inc.) was used in all measurements of H_2_O_2_ and peroxisome abundance.

In respect to the peroxisome analysis, sampling in three biological replicates was done at the maximum stress. Here, we advanced the technique to sample into the Deep 96-well-collection plates (2 ml) in liquid nitrogen to match the high-throughput format (Figure S5). The plates were placed in the Geno/Grinder®–automated tissue homogenizer and cell lyser (SPEX sample Prep, NJ, USA) for grinding leaf tissues. The assay of peroxisomes quantification was prepared and proceeded according to Fahy et al. (2017) using a specific fluorescent dye Nitro-BODIPY for staining plant peroxisomes (Landrum et al., 2010). Three technical replicates were assayed for each sample. The intensity of fluorescence signals was measured at an excitation wavelength of 490 nm and an emission wavelength of 530 nm. While protein content measured at an absorbance of 595 nm, the fluorescence intensity of peroxisome content was calculated in relation to the assigned concentration of 1 mg of the extracted protein content. To image peroxisomes, the basal leaf part was incubated for 10 min at room temperature in a freshly prepared 1 μM solutions of N-BODIPY in distilled water. Peroxisomes were imaged using resonant mode (12,000 Hz) of a Leica SP8 laser scanning confocal microscope, 512 × 512 pixels image resolution, four averages which correspond to the image acquisition rate of 0.1 s per frame.

### Scoring Polymorphism of Plant Height Alleles Using KASP Markers

The plant height was correlated with alleles at the *Rht-B1* and *Rht-D1* genes for reduced height in wheat. Single molecular markers have been developed using Kompetitive allele-specific PCR (KASP) endpoint genotyping technology which was designed based on point mutation of the plant height genes *Rht-B1* (wMAS000001) and *Rht-D1* (wMAS000002) on chromosome 4B and 4D of wheat genome (Ellis et al., 2002). These markers were listed in the casual marker catalog of wheat and KASP markers cereals database (CerealsDB). The endpoint genotyping was conducted using the Light Cycler®480 II (Roche Diagnostics Ltd. Forrenstrasse, Switzerland). The KASP assay was performed according to the LGC Genomics, Ltd. (Middlesex, UK) guide manual.

### Quantification of Peroxisome Biogenesis Genes

#### Genes Prediction and Primer Design

The homologs of *PEX11, FIS1*, and *DRP3A,3B, 5B* genes were identified in *T. aestivum*, using the known genes of *Brachypodium distachyon* as reference genes. First, referenced gene sequences were used in ViroBLAST server (https://wheat.pw.usda.gov/GG2/WheatTranscriptome/viroblast/viroblast.php) through the BLASTn tool (published wheat transcripts database) to identify wheat transcripts. In addition, wheat proteins were identified using the BLASTx against (complementary wheat proteins). Subsequently, the highly significant aligned wheat transcripts and proteins were tested against the protein database using BLASTx and BLASTp through NCBI and TAIR servers to confirm the homology to the known genes and proteins in Arabidopsis. Predicted transcripts and protein sequences were displayed in Table S2. The predicted genes were used to design primers for quantitative real-time PCR (qPCR) analysis.

#### Quantitative Real-Time PCR Experiment

The same samples that were collected for peroxisome analysis were used for examining the patterns of the mRNA expressions. The total RNA was extracted from three biological samples using RNeasy plant kit (QIAGEN) according to the manufacturer's instructions. The cDNA strand was synthesized using Maxima H Minus First Strand cDNA Synthesis Kit (Thermo Scientific, K1681). The qPCR reactions were prepared using Fast SYBR® Green Master Mix (Thermo Scientific, 4385612), and reactions were performed for each genotype and replicated in three technical repeats on Applied Biosystems® 7500 Fast Real-Time PCR System. Actin was used as a housekeeping gene. All the designed primers were listed in Table S3. Folding changes in gene expression were calculated according to Livak and Schmittgen (2001).

### Statistical Analysis

Each measurement for each genotype was done using three biological repeats. The overall mean for each parameter was calculated for all of the used genotypes. An index of response to stress was calculated as [(trait value after stress-trait value before stress)/trait value before stress ^*^100]. The analysis of variance was performed for all traits individually with all factors which were considered as fixed. All analyses were performed using the SAS-Enterprise and the SAS-JMP software (SAS Institute, Cary NC). Multiple comparisons were performed using Tukey and Dunnet's comparison tests. The relationships among individual variables were determined using Pearson's correlation coefficient (*R*^2^) and Principal Component Analysis (PCA), using a robust method. The Receiver Operating Characteristics (ROC) curves were generated to predict the probability of a true positive (sensitivity) and a false positive rate (1-specificity) for each phenological trait and genotype. Broad-sense heritability (H) for each trait was estimated under non-stressed and stressed conditions using estimated variance components (Rasmusson and Lambert, 1961). Calculating heritability was done manually based on the estimation of the phenotypic (δ^2^p) and genotypic (δ^2^g) variance according to Toker (2004), which H = $\frac{\delta 2\text{g}}{\delta 2\text{p}}$, where (δ^2^p) = δ^2^g+(δ^2^gs/s) + (δ^2^e/sr). Where “δ^2^g” is the genetic variance and “δ^2^p” is the phenotypic variance. Variance “δ^2^gs” is the genotype by environment interaction, and “δ^2^e” is the residual variance, “s” refers to the number of environments, and “r” is the number of replications. The calculated broad-sense heritability values were confirmed through the statistical application of Multivariate analysis application, MVApp (http://mvapp.kaust.edu.sa/MVApp/). Graphs were plotted using the Graph Pad Prism software.

## Results

### Phenotyping Plant Responses to Severe Drought

#### Physiological Responses

**Figure 1:**
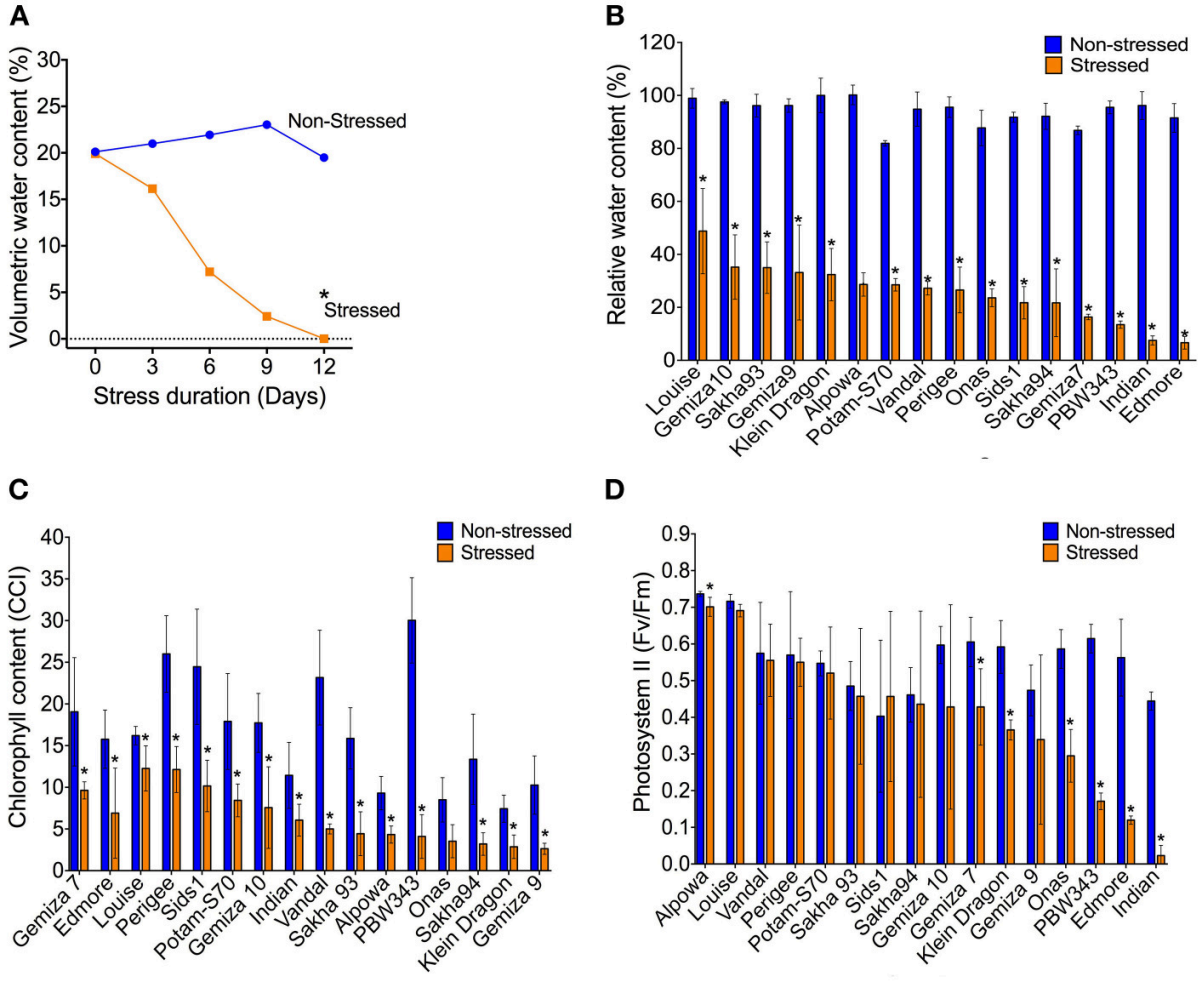
The volumetric water content of soil reached the permanent wilting point (0%) on the 12th day of water withholding **(A)**. The severe water deficiency shows a range of impacts on; the plant relative water content **(B)**, chlorophyll content **(C)**, and the photosynthetic activity **(D)**.

Withholding watering caused a progressive reduction of the VWC and the soil electrical conductivity while the soil temperature was not affected (Figure 1A; Figures S6A–C). By day 12, the VWC reached 0% in all the genotypes compared to ca. 25.4% of the watered controls. That level was considered as a point of drought stress and the phenotypic traits were measured. The reduction of the soil moisture content was accompanied by a reduction of the plant relative water content. The average of RWC for the stressed plants was almost three times lower than the control (25.4 vs. 74%; Figure S6D). The individual genotypes exhibited variability under both normal watering and drought conditions (Figure 1B). The severe drought stress caused an overall increase in the leaf temperature by 0.7°C which was associated with a dramatic reduction in chlorophyll content in all stressed plants in relation to the control (Figure 1C; Figures S6E–G). The photosystem II activity of some individual genotypes was dramatically influenced by the severe stress, such as Indian, Onas, Edmore, PWB343, Klein Dragon, Gemiza7, and Alpowa, while no change was recorded in the rest of the genotypes (Figure 1D). The total reduction of chlorophyll fluorescence (PSII) was only 15.4%, whereas the chlorophyll content was 48.9% (Table 1).

**Table 1:** Impact of prolonged drought on the traits.

**Trait category**	**Trait**	**Unit**	**Mean ± SD**	**Mean ± SD**	**Sample number**	***P*-value**	**Trait index**
			**Non-stressed**	**Stressed**			
Soil moisture	Volumetric water content	%	19.49 ± 3.47	0.16 ± 0.94	48	*P <* 0.0001	−99.17
	Electrical conductivity	ds/m (m^3^/m^−3^^*^100)	0.7 ± 0.89	0.0002 ± 0.0014	48	*P <* 0.0001	−99.97
	Soil temperature	°C	28.22 ± 2.94	28.34 ± 1.98	48	*P <* 0.8169	+0.42
Plant moisture	Relative water content	%	92.76 ± 16.75	25.41 ± 12.95	48	*P <* 0.0001	−65.36
Plant physiology	Photosystem II	Fv/Fm	0.48 ± 0.19	0.41 ± 0.22	48	*P <* 0.0382	−15.44
	Leaf temperature	°C	28.66 ± 1.79	29.40 ± 1.36	48	*P <* 0.0251	+2.58
	Chlorophyll content	CCI	14.89 ± 7.73	7.60 ± 5.43	48	*P <* 0.0001	−48.96
Plant cell	Peroxisome abundance	Relative value (AU)	123.31 ± 80.52	306.41 ± 240.61	48	*P <* 0.0001	+148.49
Plant morphology	Plant height	cm	55.12 ± 15.22	41.42 ± 25.44	48	*P <* 0.0009	−24.87
	Peduncle length	cm	23.13 ± 7.57	15.49 ± 10.51	48	*P <* 0.0001	−33.05
	Awn length	cm	5.61 ± 2.60	3.79 ± 2.74	48	*P <* 0.0012	−32.52
	Root dry weight	gm	0.69 ± 0.28	0.37 ± 0.28	48	*P <* 0.0001	−46.05
	Total root Length	cm	377.33 ± 172.17	231.89 ± 135.58	48	*P <* 0.0001	−38.54
	Survival rate	Absolute value	10 ± 0	6.37 ± 1.61	48	*P <* 0.0001	−36.25
	Recovery rate	Absolute value	10 ± 0	5.75 ± 2.79	48	*P <* 0.0001	−42.50
	Day to flower	days	44.75 ± 6.12	47.82 ± 6.90	48	*P <* 0.0295	+6.87
Plant biomass	Relative plant dry weight	gm	18.71 ± 8.88	6.80 ± 8.85	48	*P <* 0.0001	−63.68
	Standing biomass	Relative value (AU)	11.06 ± 5.64	4.20 ± 5.32	48	*P <* 0.0001	−62.00
Yield traits	Tiller number	Absolute value	55.12 ± 15.22	41.42 ± 25.44	48	*P <* 0.0001	−61.58
	Total spike number	Absolute value	11.52 ± 4.48	4.37 ± 4.61	48	*P <* 0.0001	−62.03
	Number of productive tillers	Absolute value	10.35 ± 4.11	3.89 ± 4.58	48	*P <* 0.0001	−62.37
	Spike weight	gm	11.33 ± 6.28	3.74 ± 5.11	48	*P <* 0.0001	−66.95
	Grain number	Absolute value	245.44 ± 131.56	95.85 ± 130.88	48	*P <* 0.0001	−60.95
	Grain weight	gm	8.29 ± 4.52	2.71 ± 3.65	48	*P <* 0.0001	−67.33

*Means and standard deviation were calculated for each trait. Trait index is the reduction or increases in the trait value of the stressed plants compared with the non-stressed. Means at 95% confidence level were taken based on the one-way ANOVA test*.

#### Genotyping Plant Height Alleles

The genetic identification using KASP markers of the plant height alleles of *Rht-B and Rht-D genes* confirmed four gene descriptions (Table 2). The tall genotypes (Onas, Indian, and Edmore) was identified by the combination of Rht-B1a/Rht-D1a. The semi-dwarf genotypes were classified into two groups; semi-dwarf group A *(Rht-B1b/Rht-D1a)* included Klein Dragan, PBW343, Gemiza10, Gemiza9, Gemiza7, Sakha94, and Sids1. While, Alpowa, Louise, Potam-S70, Vandal, and Sakh93 genotypes were characterized as semi-dwarf group B *(Rht-B1a/Rht-D1b)*. Perigee is the only dwarf genotype *(Rht-B1b/Rht-D1b)*.

**Table 2:** Results of the Kompetitive Allele-Specific PCR (KASP) genotyping of Rht-B1 and Rht-D1.

**Genotype**	**Alleles components**	**Plant height description**
Onas	Rht-B1a/Rht-D1a	Tall
Indian	Rht-B1a/Rht-D1a	Tall
Edmore	Rht-B1a/Rht-D1a	Tall
Klein dragon	Rht-B1b/Rht-D1a	Semi dwarf (A)
PBW343	Rht-B1b/Rht-D1a	Semi dwarf (A)
Gemiza10	Rht-B1b/Rht-D1a	Semi dwarf (A)
Gemiza9	Rht-B1b/Rht-D1a	Semi dwarf (A)
Gemiza7	Rht-B1b/Rht-D1a	Semi dwarf (A)
Sakha94	Rht-B1b/Rht-D1a	Semi dwarf (A)
Sids1	Rht-B1b/Rht-D1a	Semi dwarf (A)
Aplowa	Rht-B1a/Rht-D1b	Semi dwarf (B)
Louise	Rht-B1a/Rht-D1b	Semi dwarf (B)
Potam-S70	Rht-B1a/Rht-D1b	Semi dwarf (B)
Vandal	Rht-B1a/Rht-D1b	Semi dwarf (B)
Sakha93	Rht-B1a/Rht-D1b	Semi dwarf (B)
Perigee	Rht-B1b/Rht-D1b	Dwarf

#### Peroxisome Proliferation Response

**Figure 2:**
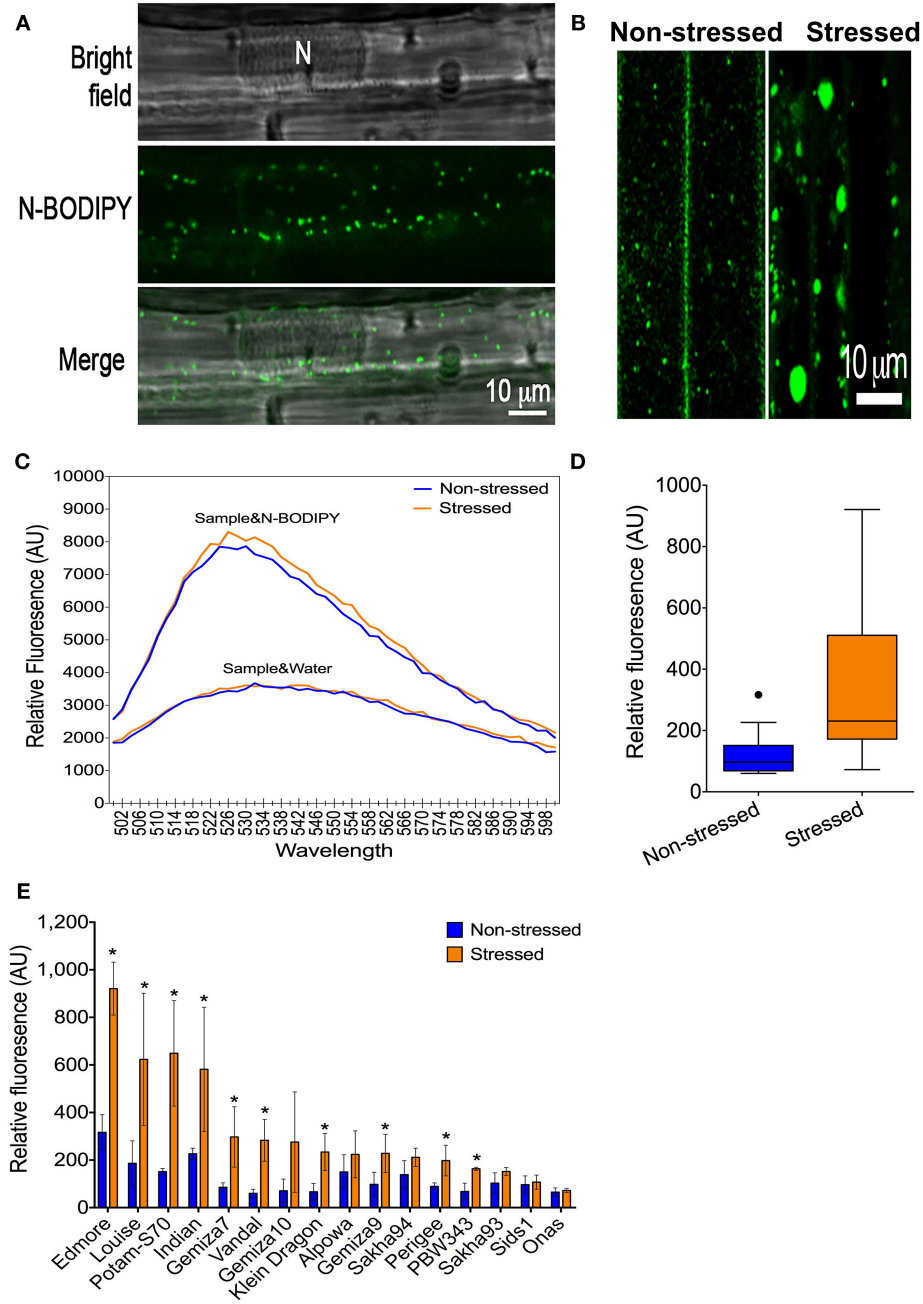
A phenotype of peroxisome abundance under severe drought: **(A)** Staining of peroxisomes in leaf-epidermis cells using N-BODIBY dye. **(B)** A phenotype of enlarged peroxisomes under drought. **(C)** The emission spectra of the total protein extract alone or combined with N-BODIPY fluorescent dye in the non-stressed sample and stressed sample. **(D)** The overall gain of the average of peroxisome abundance in non-stressed and stressed leaves. **(E)** Impact of drought on peroxisome proliferation in individual genotypes. All blue (lines or bars), and orange (lines or bars) refers to non-stressed and stressed samples, respectively.

N-BODIPY specifically stained peroxisomes in wheat leaf epidermis cells (Figure 2A) and exhibited a dynamic behavior that was orchestrated by rapid movements and static phases in normal conditions (Figure S7, see the Movie in the Supplementary Materials). Peroxisomes were larger in the leaf epidermis cells from the plants that were subjected to the severe drought stress in comparison to those with non-stressed cells (Figure 2B). The peroxisome quantification technique was applied to phenotyping the population in a high-throughput format (Figure S5). Excitation of 0.5 μM N-BODIPY in the total leaf protein extract at 490 nm resulted in an emission spectrum with a maximum at 526 nm (Figure 2C). No fluorescence has been detected in the absence of the proteins extract, while the protein extracts alone produced a background fluorescence that lacked a definite peak. Extracts from drought-stressed leaves produced higher fluorescent signal than the non-stressed extracts at the same emission spectra in the presence of N-BODIPY only (Figure 2C). The drought promoted the overall peroxisome proliferation in the population (Figure 2D), despite the significant variation amongst the individual genotypes, while no change in peroxisome abundance was detected in Onas genotype (Figure 2E).

#### Morphological Parameters

**Figure 3:**
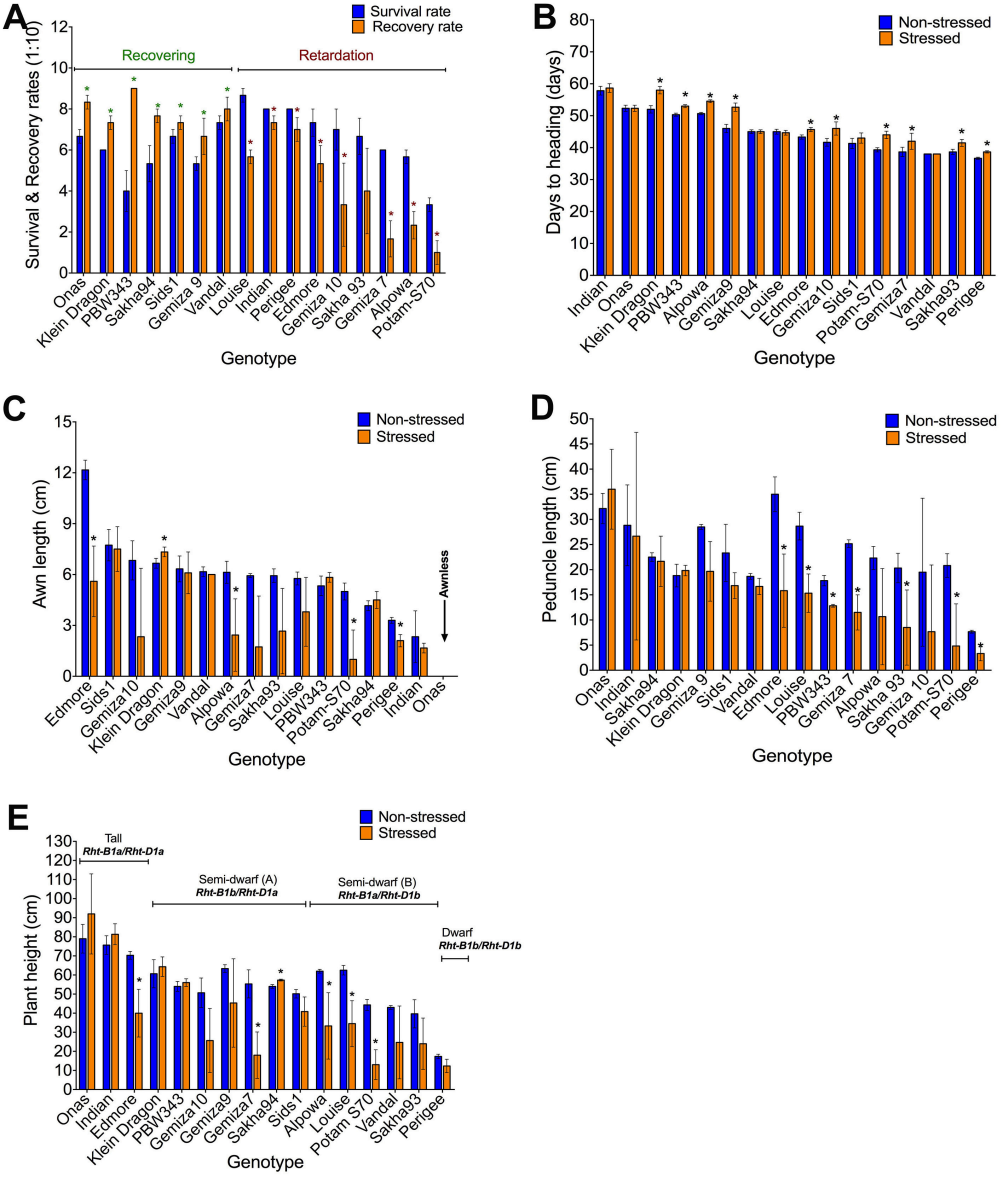
Impact of severe drought on the obtained morphological traits. Variation in the genetic response of survival and recovery rates, the response of genotypes was classified into recovering and retarding responses **(A)**, days to heading **(B)**, awn length **(C)**, peduncle length **(D)**, and plant height alleles **(E)**.

**Figure 4:**
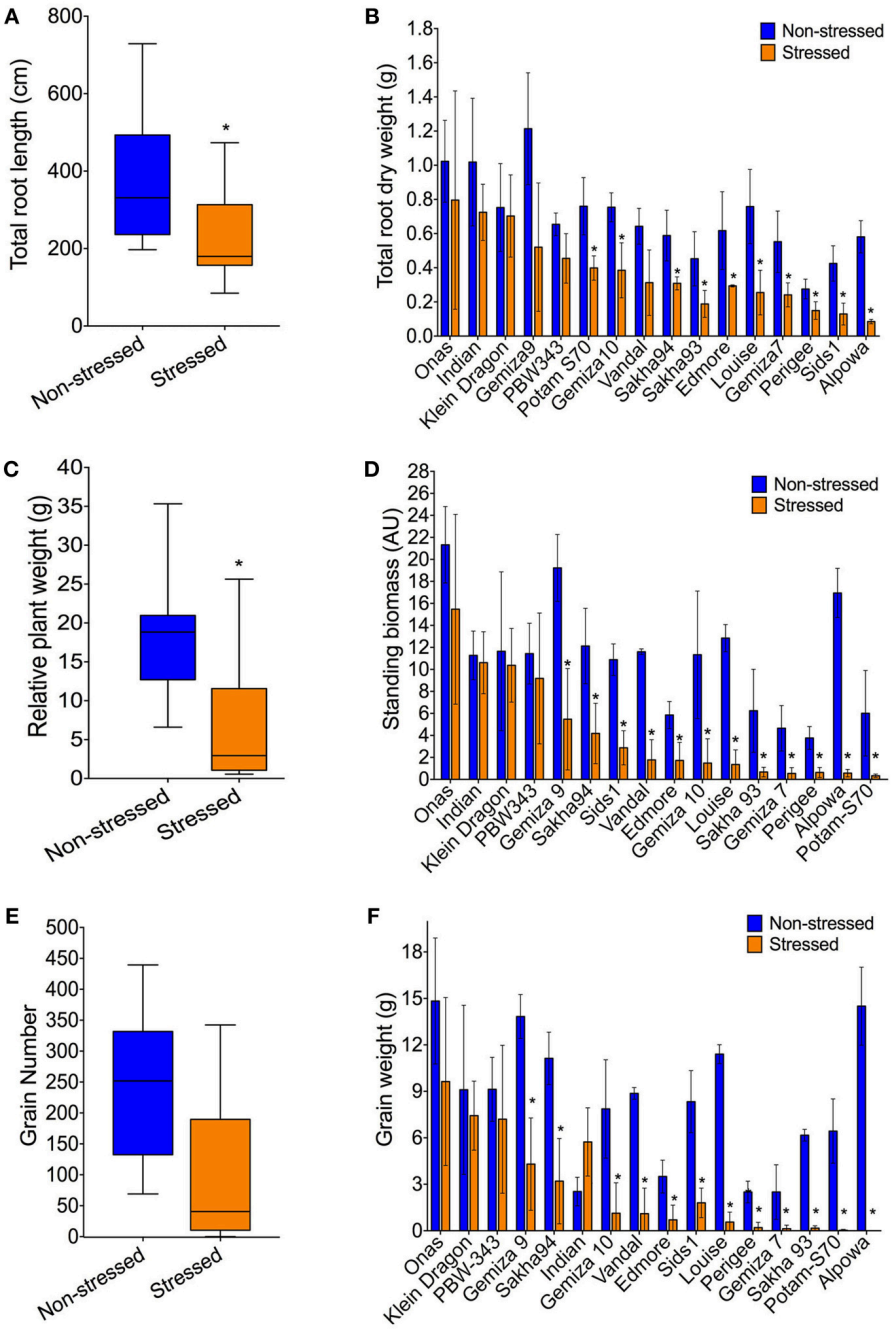
Impact of severe drought on: Root traits including the reduced overall mean of total root length biomass **(A)**, and the individual total root dry weight of genotypes **(B)**. Plant biomass through the average of relative plant weight **(C)**, and the genetic variation of the standing biomass **(D)**. The overall reduction of grain number **(E)**, and the variable response of grain yield represented by grain weight among the genotypes **(F)**.

Watering was resumed after sampling (phenotyping) on day 12 and plant recovery has assessed on day 24 (Figure S3). It was observed that the total reduction was 36.3% in the survival rate and 42.5% in the recovery rate (Table 1). The stress symptoms persisted or became more severe in nine genotypes: Indian, Perigee, Louise, Edmore, Gemiza10, Gemiza7, Sakha93, Potam-S70, and Alpowa. The remaining genotypes exhibited signs of recovery (Figure 3A). Severe drought stress caused heading delay in 10 genotypes by an average of 7 days (Figure 3B). The awn length, peduncle length, and plant height were significantly reduced under stress by 32.52, 33.05, and 24.87% respectively (Table 1). Several genotypes (Sids1, Gemiza9, Vandal, PBW343, Sakha94, and Indian) retained normal awn length under stress while a reduction was observed in the remaining genotypes with the exception of the awnless genotype Onas (Figure 3C). The peduncle length was significantly reduced in all genotypes with the exception of Onas, Sakha94, Klein Dragon, Gemiza 9, Sids1, and Vandal (Figure 3D). The impact of prolonged drought on the plant height varied across the genotypes (Figure 3E). The only double dwarf (Perigee) did not show a significant height reduction due to stress treatment. The impact of drought on plant height was not consistent across the tall genotypes. Plant height of Edmore reduced, while no significant change was detected in Onas and Indian. A general reduction in plant height was observed among the genotypes of semi-dwarf B group. While no significant reduction was shown in the semi-dwarf A group except Gemiza7. The overall means of total root length was reduced in the drought-stressed plants (Table 1 and Figure 4A), while no significant inhibition in root dry weight was detected in Onas, Indian, Klein Dragon, Gemiza9, and PBW343 genotypes (Figure 4B; Figure S8).

#### Biomass and Grain Yield After Stress Recovery

**Figure 5:**
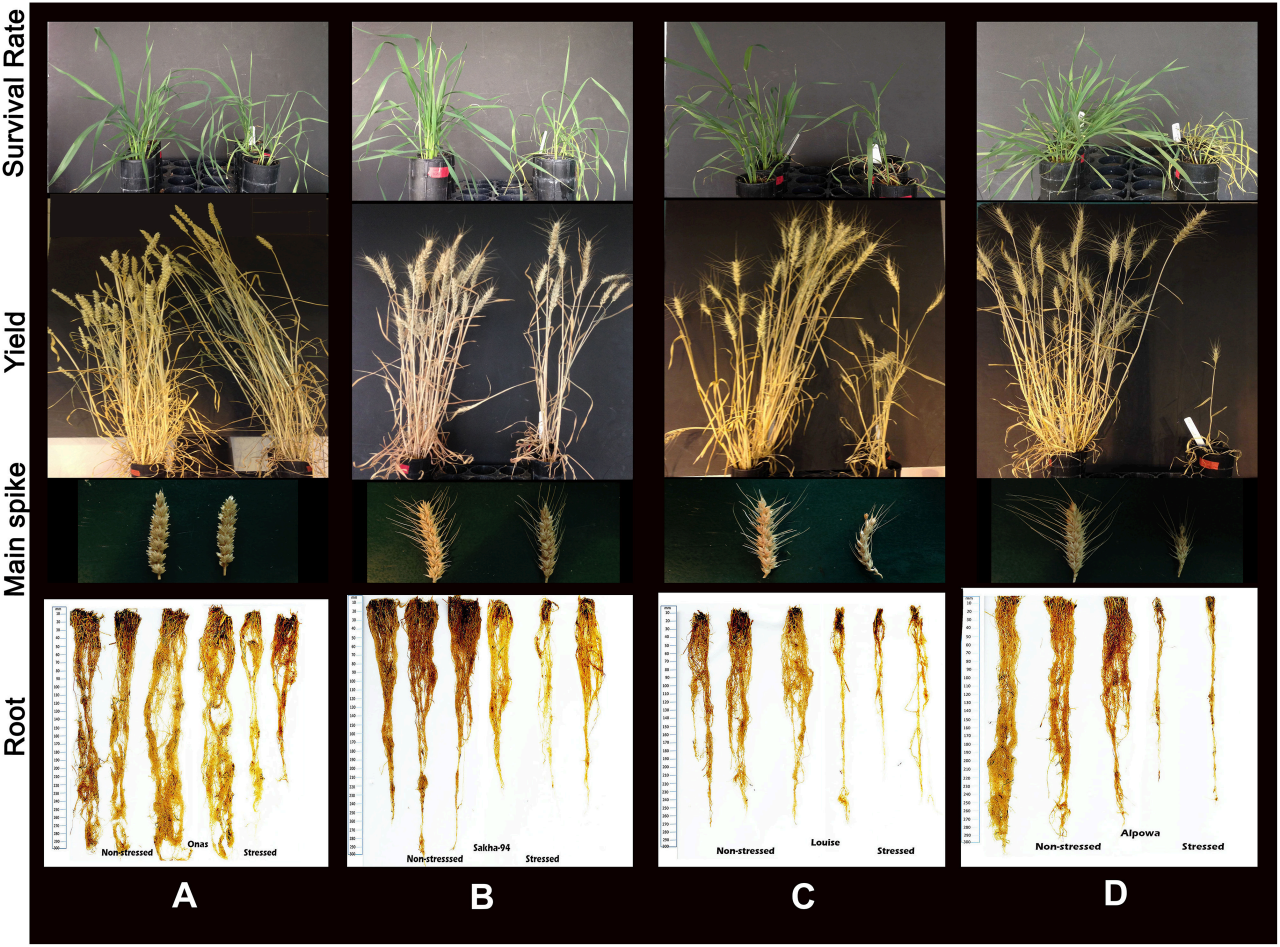
Representative images of different response to the drought. **(A)** An adaptive plant represents Onas, associated with the minimal impact of drought. **(B)** Moderate adaptation by the Sakha94, smaller spikes with shorter awns, and shorter roots. **(C)** Limited adaptation of Louise, limited recover, a significant loss in yield and spike shrinkage and rolling associated with significant reduction in root biomass. **(D)** Susceptibility to drought by Alpowa, abnormal spike, and lack of yield and dead roots.

Consistent with the inhibition of plant growth and photosynthetic traits under the stress, biomass yield was affected (Table 1). The overall mean of relative plant dry weight significantly reduced (Figure 4C). The reduction percentage of the relative plant dry weight and standing biomass was 63.68 and 62%, respectively (Table 1), and a significant correlation was found between both traits (*R*^2^ = 0.79). The reduction in plant biomass was scored in most of the genotypes except Onas, Indian, Klein Dragon and PBW343 (Figure 4D). Consequently, after recovering from severe drought. All grain yield traits were inhibited as follows: tiller number (61.58%), total spike number (62.03%), number of productive tillers (62.37%), total spike weight (66.95%), grain number (60.95%), and grain weight (67.33%) (Table 1 and Figures S9A–D). We found that all yield traits including plant biomass are highly correlated with each other (Table S4). Thus, grain weight was selected as the representative trait to assess genotypes performance or yield fitness in response to the stress. Yield traits of Onas, Indian, Klein Dragon, and PWB343 were not significantly affected by the drought. In contrast, the yield of Gemiza10, Sids1, Edmore, Vandal, Perigee, and Louise was significantly reduced. The lowest yield performance genotype under our experimental conditions was Alpowa, which failed to develop productive spikes (Figure 4F). Various levels of survival prevailed under the prolonged drought that was displayed in plant yield, awns, and roots (Figures 5A–D).

### Traits Analysis

#### Traits Heritability

The broad sense heritability was calculated to all the phenological traits under both non-stressed and stressed conditions (**Table 4**). In general, the heritability decreased in most of the traits under stressed conditions, whilst it increased in 9 traits out of 24 as (number of productive tillers, spikes weight, peroxisome abundance, relative water content, chlorophyll content, leaf temperature, PSII, and survival rate). Here the overall heritability of grain yield reduced from 89.72% in non-stressed conditions to 81.86% in stressed conditions.

#### Trait Correlation and the Principal Component Analysis (PCA)

**Figure 6:**
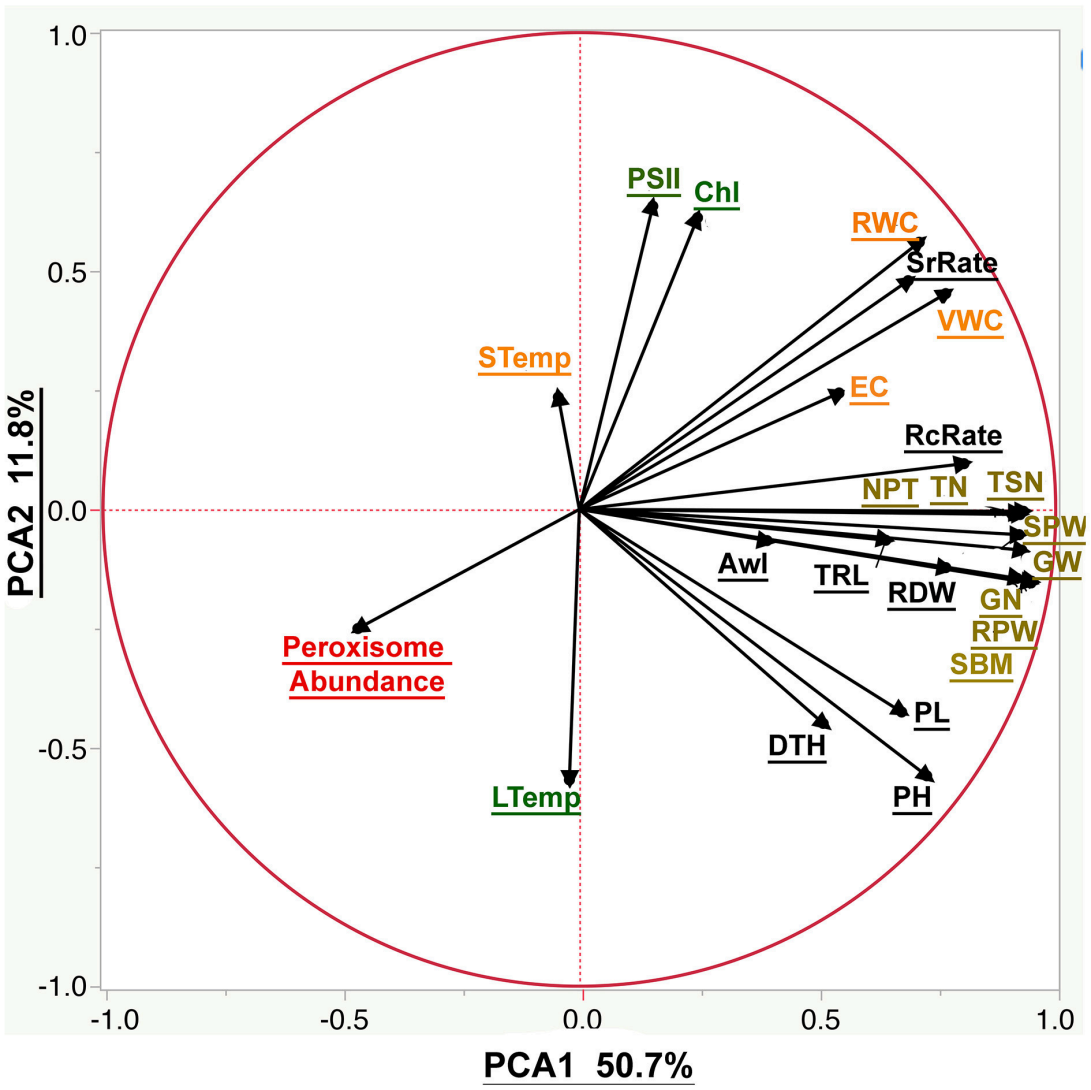
The biplot of the principal component analysis (PCA) of the phenotypic traits. The PCA was plotted using all the measured phenological traits. Moisture parameters include volumetric water content (VWC), electrical conductivity (EC), soil temperature (STemp), and the relative water content (RWC). Morphological parameters include plant height (PH), peduncle length (PL), root dry weight (RDW), total root length (TRL), relative plant dry weight (RPW), standing biomass (SBM), awn length (Awl), days to heading (DTH), the survival rate (SrRate), and recovery rate (RcRate). Yield components include tiller number (TN), total spike number (TSN), number of productive tillers (NPT), total spike weight (SPW), grain number (GN), and grain weight (GW). Cellular parameter by peroxisome abundance. Photosynthetic parameters are represented by the chlorophyll content (Chl), photosystem II (PSII) and leaf temperature (LTemp).

Our phenotypic analyses revealed two trends: (1) variable response to severe drought stress amongst the 16 genotypes; and (2) different amplitude of the response to drought amongst the 24 traits. To determine the patterns in the correlations among the individual traits, the correlation structure, supported with significance values, was calculated (Table S4) and a principal component analysis (PCA) was plotted (Figure 6).

Correlating the phenological traits to grain yield is our axis to evaluate the suitability of the phenological traits to phenotype plant response to drought. The correlation between grain yield and recovery rate was 0.64 and more scattered with the survival rate 0.5 (Scatterplots provided in the Table S4). Grain yield correlated more strongly with root dry weight (*R*^2^ = 0.74) than with root length (*R*^2^ = 0.55), or awn length (*R*^2^ = 0.27). Furthermore, peroxisome abundance was negatively correlated with soil moisture, plant water content, root traits and grain yield (*R*^2^ = −0.42, −0.49, −0.29, and −0.39, respectively). The weakest correlation was found between yield and chlorophyll content (*R*^2^ = 0.14) as well as photosystem II activity (*R*^2^ = 0.1). That is to say that leaf and soil temperature didn't correlate with the yield traits. The plant height scored limited correlation with the survival rate during the drought (*R*^2^ = 0.29) and stronger correlation with the recovery rate, grain yield, peduncle height, standing biomass, and root dry weight (*R*^2^ = 0.58, 0.67, 0.8, 0.73, and 0.62, respectively). Also, a significant negative correlation with peroxisome abundance (*R*^2^ = −0.22) was recorded (Table S4).

The PCA analysis was used to reduce data dimensionality among all the traits (Figure 6). PCA 1 was the most explanatory component that explained 50.7% of the explained variation. PCA 1 correlates positively to traits related to yield components, plant recovery, and plant height while peroxisomal abundance has large negative loadings on PCA components. Moreover, PCA 2 explained a much smaller portion of observed variation (11.8%), corresponding mainly to chlorophyll fluorescence and abundance.

#### The Receiver Operating Characteristics (ROC) Test

**Figure 7:**
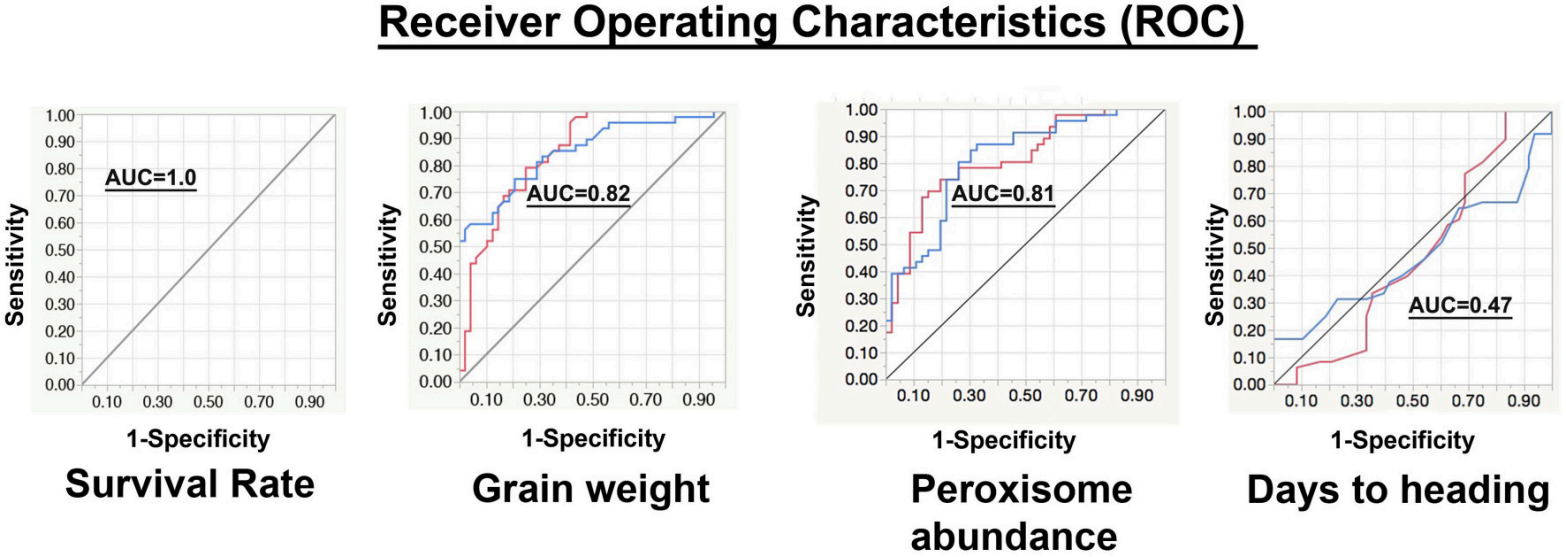
Representation of the receiver operating characteristics (ROC). In the chart, the red curve corresponds to the non-stressed response and the blue curve represents the stressed response. The y-axis refers to the sensitivity or (true positive rate), while the x-axis is the false positive rate (1-specificity). The area under the curve (AUC) of survival rate, grain weight, peroxisome abundance, and days to heading are 1.00, 0.82, 0.81, and 0.47, respectively.

Values of the area under the curve (AUC) of all the phenotypic traits were calculated (Table 3). Based on the AUC values the ROC plots (Table 3 and Figure 7) of the phenological traits shown: (1) most of the phenological traits ranged between 1 and 0.75, (2) plant height, PSII, awns length, and leaf temperature was close to the sensitivity axis (0.69, 0.69, 0.67, 0.61), (3) the lowest sensitivity values scored by soil temperature (0.49) and days to heading (0.47).

**Table 3:** Analysis of the area below the operating characteristics analysis (ROC), the area under the curve (AUC) for each trait and per trait categories under non-stressed, and stressed conditions.

**Trait category**	**AUC value per trait category**	**Traits**	**Abbreviation**	**AUC value per trait**
Soil moisture	1.00	Volumetric water content	VWC	1.00
		Electrical conductivity	EC	0.99
		Soil temperature	STemp	0.49
General morphology	1.00	Survival rate	SrRate	1.00
		Recovery rate	RcRate	1.00
Plant moisture	0.980	Relative water content	RWC	0.98
Plant height	0.765	Plant height	PH	0.69
		Peduncle length	PL	0.76
Awn Length	0.673	Awn length	Awl	0.67
Root morphology	0.829	Total root dry weight	RDW	0.83
		Total root Length	TRL	0.78
Heading time	0.469	Day to heading	DTH	0.47
Biomass	0.859	Relative plant dry weight	RPW	0.85
		Standing Biomass	SBM	0.83
Yield components	0.917	Tiller number	TN	0.87
		Total spike number	TSN	0.87
		Number of productive tillers	NPT	0.86
		Total Spike weight	SPW	0.85
		Grain number	GW	0.82
		Grain weight	GN	0.85
Photosynthetic	0.791	Photosystem II	PSII	0.69
		Leaf temperature	LTEMP	0.61
		Chlorophyll content	Chl	0.79
Cellular	0.815	Peroxisome abundance	PeroxAbun	0.81

**Table 4:** Broad-sense heritability percentage of the phenological traits under per trait categories under non-stressed and stressed conditions.

**Trait category**	**Traits**	**Abbreviation**	**Broad-sense heritability (%)**	
			**Non-stressed**	**Stressed**
Soil moisture	Volumetric water content	VWC	90.02	0
	Electrical conductivity	EC	53.11	0
	Soil temperature	STemp	99.63	99.66
General morphology	Survival rate	SrRate	65.75	85.49
	Recovery rate	RcRate	65.75	87.40
Plant moisture	Relative water content	RWC	77.85	78.17
Plant height	Plant height	PH	96.84	89.66
	Peduncle length	PL	82.26	68.91
Awn Length	Awn length	Awl	97.83	80.69
Root morphology	Total root dry weight	RDW	77.36	65.99
	Total root Length	TRL	93.01	69.89
Heading time	Day to heading	DTH	98.16	66.82
Biomass	Relative plant dry weight	RPW	86.48	80.23
	Standing Biomass	SBM	85.60	83.50
Yield components	Tiller number	TN	89.87	82.44
	Total spike number	TSN	86.55	82.52
	Number of productive tillers	NPT	83.62	84.67
	Total Spike weight	SPW	58.08	80.16
	Grain number	GW	89.72	81.86
	Grain weight	GN	86.78	81.06
Photosynthetic	Photosystem II	PSII	65.83	80.54
	Leaf temperature	LTEMP	87.10	90.38
	Chlorophyll content	Chl	71.83	88.79
Cellular	Peroxisome abundance	PeroxAbun	84.49	88.47

### Dynamics of Peroxisome Proliferation During a Time-Course of Drought

#### Dynamic Changes in Phenological Parameters

**Figure 8:**
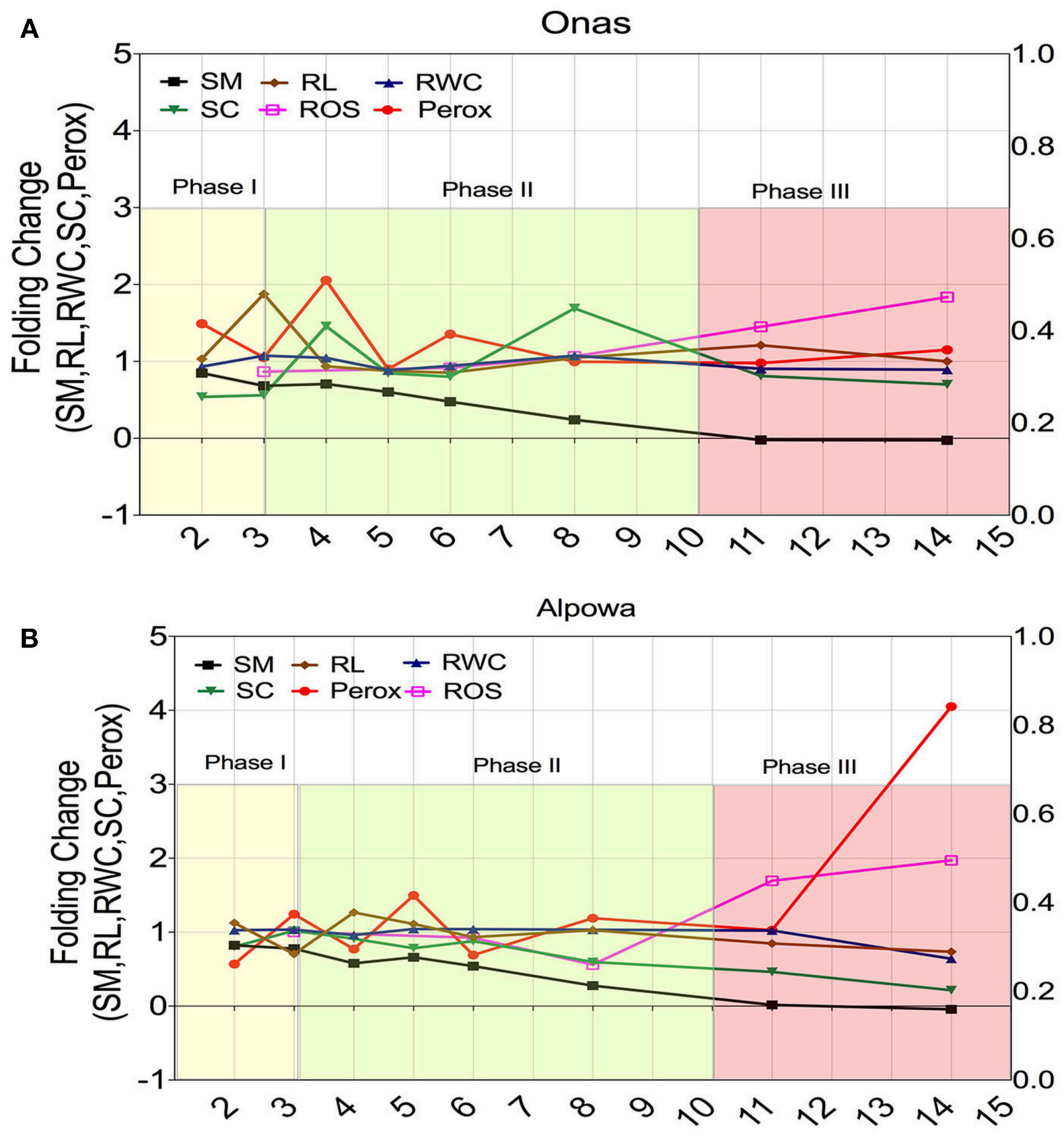
Dynamic changes of the; soil moisture (SM), root length (RL), relative water content (RWC), stomatal conductance (SC), peroxisome abundance (Perox), and ROS content (ROS) during three successive phases of drought in a selection of two genotypes that had shown contrasting phenotypic performance under drought. **(A)** The dynamic changes in the tolerant genotype (Onas), while **(B)** illustrates the dynamic changes in the susceptible genotype (Alpowa). The y-axis represents the folding change of each trait in relation to the non-stressed groups, while the x-axis represents the time points (days) during drought phases. The beginning of phase II discriminated the dynamics of peroxisome abundance and stomatal conductance of the tolerant response, while the end of phase III discriminated the susceptible response.

Two contrasting genotypes were selected based on their adaptive performance; the highest adaptive genotype (Onas) (Figure 8A) and the lowest adaptability (Alpowa) (Figure 8B) to monitor the dynamic changes of six phenological traits (soil moisture, peroxisome abundance, RWC of leaves, stomatal conductance, root length, and ROS) through three successive phases of drought. Folding change of the phenological traits was estimated in relation to the values under the non-stressed. During phase I, a stomata closure event was signaled by a slight reduction in soil moisture only in the tolerant genotype Onas with an absence of any change in RWC or ROS content. Early peroxisomes proliferation was detected during phase II in Onas, (2-folds) and 1.5-fold in Alpowa at day 4 and 5, respectively. By extending the drought till day 6, 1.3-fold of peroxisome abundance was observed only in Onas. At the end of phase II (8th day), the soil moisture content reached 0.25-fold. That associated with a dramatic reduction in stomatal conductance and ROS content was observed in Alpowa vs. an elevation of the stomatal conductance in Onas. Day 11 and day 14 in phase III were assigned to record severe and prolonged drought conditions, respectively. Significant accumulation of ROS content was detected during phase III in both genotypes. In Alpowa, the ROS accumulation was associated with a great abundance of peroxisomes and a dramatic reduction in stomatal conductance values as well as RWC and root development. On the other hand, Onas had no changes in peroxisome abundance, RWC, and root development with a slight reduction in stomatal conductance.

#### Dynamic Changes in Gene Expression of Peroxisome Biogenesis Genes

Three-time points, the 3rd, 8th, and 14th days, were selected to represent the successive phases of drought for quantifying the gene expression of the peroxisome biogenesis genes in both genotypes. All transcripts values were normalized in relation to the level of expression in the untreated samples.

**Figure 9:**
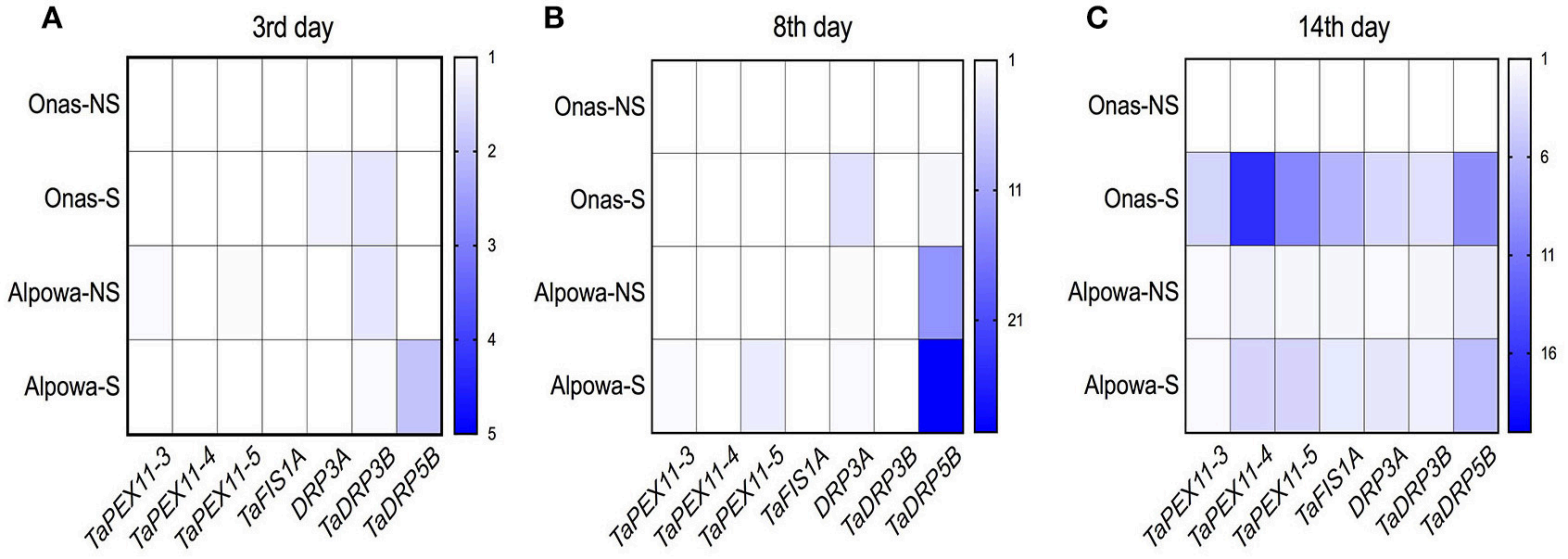
Heatmaps display the folding changes in expression of wheat peroxisome biogenesis genes, peroxisomal factor11 genes (TaPEX11.3, TaPEX11.4, TaPEX11.5), Mitochondrial fission 1A (FIS1A), and dynamin-related proteins (TaDRP3A, TaDRP3B, TaDRP5B) after **(A)** 3, **(B)** 8, and **(C)** 14 days of withholding watering. The y-axis represents the genotypes per treatment (NS, non-stressed; S, stressed), while the x-axis represents the tested genes. Three technical replicates were used for each of the three biological replicates.

At the 3rd day, as shown in Figure 9A, a slight up-regulated expression of *TaDRP3A* and *TaDRP3B* genes with 1.2-, 1.6- folds, respectively were found in Onas under stressed conditions while 1.9-folds of *TaDRP5B* gene was up-regulated in stressed Alpowa. Meanwhile, we noticed the up-regulated expression of *TaDRP3B* gene in Alpowa under the normal conditions. A unique upregulated expression of *TaFIS1A* gene and *TaPEX11-5* gene was exclusively found in stressed Onas and Alpowa, respectively after 8 days of drought (Figure 9B). In the meantime, the expression of *TaDRP5B* gene has appeared in Alpowa genotype only, but the gene was up-regulated in stressed conditions more than non-stressed conditions gene expression. By reaching the most stress severity at day 14 (Figure 9C), all tested genes were up-regulated at both genotypes under stressed conditions. Interestingly, more gene expression was detected in stressed Onas than Alpowa, especially *TaPEX11s, TaFIS1A*, and *TaDRP5B*. However, the peroxisome content was lower than Alpowa.

## Discussion

The most significant pathway for breeders is to enhance the plant yield per area, but the negative trade-off of traits is a barrier (Blum, 1996; Ribaut, 2006; Passioura, 2007, 2010; Tuberosa, 2012). Drought stress causes a reduction in grain yield, and heritability of grain yield under drought stress may reach 17.1% compared with 54.5% under normal conditions (Mathew et al., 2018). In the present study, several questions were investigated: (1) to assess the possibility of using a reduced and robust trait model for phenotyping the plastic plant response to drought, (2) to correlate peroxisome abundance to other traits for testing its suitability to be used as a cellular trait and establishing a high-throughput technique for quantification, (3) to analyze the differential dynamics of the adaptive response vs. the non-adaptive response under drought. Prioritizing the tested phenological traits under a plastic developmental stage as tillering stage (Sanad et al., 2016) is essential to provide accurate identification of the adaptive response. The adaptive response is controlled by several mechanisms, which may vary among certain genotypes that can develop certain mechanisms for adaption (Shinozaki et al., 2015). These adaptive mechanisms are promoted by a complex response of phenological traits, which is important to be analyzed at different time points considering the genetic variation (Chen et al., 2016).

The study highlights the importance of some phenological traits that had less attention in previous studies for phenotyping drought tolerance. For example, we found that the recovery rate, awn length, and plant height alleles may have an impact on the genotype selection of drought tolerance. Previous studies stated that plant height and peduncle length traits have been used for breeding drought-tolerant spring wheat (Okuyama et al., 2005; Heidari et al., 2012), and the heritability of plant height was 79.6% under drought (Mathew et al., 2018) and 89.66% in the current study. Although the heritability decreased under stress, it is still recording high heritability in presence of large genetic variability in plant height alleles. Which indicates plant height trait seems to have least influenced by environment The semi-dwarf wheat genotypes have been observed to be amongst the highest producers under intensive irrigation (Donmez et al., 2001; Brancourt-Hulmel et al., 2003), while taller genotypes have some advantages in semi-arid regions (Richards, 1992; Ismail and Hall, 2000). Our results revealed that drought susceptibility was more frequent among the semi-dwarf genotypes carrying Rht-B1a/Rht-D1b combination of alleles. However, among our population, no sufficient evidence was found to indicate the association between specific allele combination of plant height and plant yield.

Reporting the benefits of the awnless wheat is controversial. Awn surface area has been previously correlated with grain yield (Teare and Peterson, 1971). Awn length was suggested as a criterion in breeding programs because of their role in spike transpiration (Pask et al., 2012) and photosynthesis (Monneveux et al., 2012). Yet, other studies report that “awnless wheat” can benefit grazing, reducing disease, frost susceptibility, and has potentials to reduce pre-harvest sprout phenotype (King and Richards, 1984; Rebetzke et al., 2016). Also, in favorable environments, awnless wheat is observed with comparable yield and quality grains to the awned varieties (Rebetzke et al., 2016). In barley, higher grain yield is not associated with higher awn area (Hosseini et al., 2012). According to our findings, it is plausible to argue that higher transpiration in genotypes with long awns can limit survival to drought stress. Indirect selection for awn length may help in breeding drought tolerance.

Judging genotypes for drought adaptability based on their capabilities to be drought-resistant and drought-recovery (Chen et al., 2016). In the present study, scoring the recovery rate after re-watering was important because some genotypes after showing drought-resistance somehow under drought, the performance retarded after re-watering. Also, increasing the heritability in stressed conditions 85.49% compared with 65.75% in non-stressed conditions leads to the suitability of this trait for genetic selection.

Drought-resistance consists of three main strategies, drought-escape, drought-avoidance, and drought-tolerance (Chen et al., 2016). Plants can escape water shortage occurring late in the growing season through early flowering (Tardieu, 2013). Here, days to heading has scored to identify the genotypes that used drought-escape as a mechanism and if it is associated with drought-adaptability and investigate the potential of this trait for genetic selection. The results showed that drought-escape mechanism was associated with medium to poor performance genotypes. Also, the output results of the PCA, heritability and ROC analysis demonstrated fewer potentials for phenotyping drought-adaptability. However, an escaping strategy can be taken by certain genotypes and be a component of drought-resistance, but not necessary drought-tolerance or drought-recovery (Chen et al., 2016). Thus, it is suggested that the indirect use of days to heading, plant height, and root biomass traits is necessary for improving drought tolerance (Mathew et al., 2018).

Drought-avoidance is a major mechanism to achieve drought-adaptability (Chen et al., 2016), which is the capability of a genotype to maintain water content balanced with a reduction of soil moisture (Levitt, 1980; Basu et al., 2016). Accessing soil moisture provides a powerful tool to indicate drought state and evaluate genotype capability for avoiding drought through the rooting system. Root traits such as root dry weight and root system morphology are known to be essential for drought adaptation (de Dorlodot et al., 2007; Trethowan and Mujeeb-Kazi, 2008; Sharma et al., 2011; Comas et al., 2013), especially in C4 plants (Lopes et al., 2011). The current results demonstrated that recovered genotypes with comparable root biomass have limited peroxisome proliferation under severe drought. For instance, Mathew et al. (2018) argue that the heritability value of the root biomass was 77.8% under drought stress vs. 79.3% under normal conditions. In the present study, the reduction in heritability reached an average of 68% to indicate that those root traits have potentials for genotype selection. In practice, accurate evaluation of root architecture and tracing root traits through breeding programs is still technically challenging and not compatible with the high-throughput format.

Furthermore, maintain RWC in plants is part of the mechanism of drought-avoidance. Suitability of RWC for phenotyping responses to both drought and desiccation in crops was stated (Pammenter and Berjak, 2000; Farrant et al., 2004; Oliver et al., 2010). The prolonged and severe drought leads to a 40% reduction of RWC (Barrs and Weatherley, 1961), and subsequent inhibition of photosynthesis due to chloroplast shrinkage and higher intracellular solute concentration (Kaiser, 1982). Although RWC is considered a robust trait, it remains laborious, hence hardly applicable to high throughput settings. The RWC correlated with survival and recovery rates, as well as grain yield which reflected plant capability to resume development after re-watering. It is known that certain plants may try to balance between the water uptake and loss under drought (Basu et al., 2016). In this study, the adaptive response was dictated during drought phases to distinguish between a balanced response in the tolerant genotype (Onas) vs. a passive response followed by the tissue death in the susceptible genotype (Alpowa) at phase III. In addition, the higher heritability under drought confirms that RWC is a genetic-based trait.

Photosynthetic parameters (i.e., chlorophyll content and photosystem II activity) have secondary impacts that result from the oxidative stress and have been widely used for phenotyping the effect of the drought on plants or the identification of drought-tolerant (Lawlor and Cornic, 2002; Flexas et al., 2004; Chaves et al., 2009). The popularity of both these parameters relates to their potential in integrating complex changes in plant physiology because they are associated with direct or indirect photosynthetic changes in stomata and the mesophyll cells (Flexas et al., 2004) as well as photosynthetic metabolism (Lawlor and Cornic, 2002). In this vein, the present results showed that the correlation between photosynthetic parameters scored a high degree of variability among genotypes with weak correlation with grain yield and recovery rate. Instead, the heritability increased significantly under drought. Subsequently, the traits are less influenced by the environment and can be used for selection. Nevertheless, there are principal differences between the role of photosynthetic parameters for the survival and adaptation strategies. PSII activity cannot be used to discriminate moderate and severe water stress of wheat seedlings (Lu and Zhang, 1999). Moreover, drought stress impacts the photosynthetic capacity and the diffusion of CO_2_ in the leaves by decreasing the conductance of the stomatal and mesophyll, but the diffusive resistance limits the photosynthetic activity (Flexas et al., 2004). The association between photosynthetic activity and drought tolerance remains debatable because of the high complexity of photosynthetic response to drought (Pfannschmidt, 2003; Flexas et al., 2004; Chaves et al., 2009). Our findings agree with Wang et al. (2010) that high variability of photosynthetic parameters under drought ultimately reduces their correlation with yield.

The second experiment that monitored closely six parameters (soil moisture, RWC, stomatal conductance, peroxisome abundance, and ROS content) investigated the dynamic changes of phenological traits during three phases of drought. Indeed, that was essential to understand the mechanistic traits and distinguish the adaptive patterns using two genotypes that have contrasting adaptive performances. Our results showed that it is not necessary that the limited water deficiency during the early drought phases impact the RWC. Stomata closure is an event promoted by hormone signals of ABA and responds to the reduction of soil moisture more than the RWC. Plants survive using that trade-off strategy of managing water loss (Pirasteh-Anosheh et al., 2016), which influence the photorespiration more than the photosynthesis (Nobel, 2009) and correlates negatively with the water use efficiency (WUE) under drought (Edwards et al., 2012). The changes of the stomatal conductance are treatment dependent manner (Galmés et al., 2013) and the tolerant plant can control the stomata for achieving better carbon fixation and photosynthesis as well as improving WUE (Pirasteh-Anosheh et al., 2016). Usually, during the advanced phases of drought, a burst of jasmonic acid (JA) may lead to accumulations of abscisic acid (ABA) which stimulates the plant response in citrus roots (de Ollas et al., 2013). The production of H_2_O_2_ is promoted by Methyl jasmonate (MJ) and ABA in guard cells to induce stomata closure (Suhita et al., 2004). As a result of ROS accumulation in the plant cell, the cellular response is the initial plant response to be recognized (Shinozaki et al., 2015) particularly, peroxisome proliferation, and estimating that response was a challenge. In the meantime, our high-throughput quantitative technology for phenotyping peroxisome proliferation helped in filling this gap especially for the first-time during drought phases. Peroxisomes play a key role in maintaining cellular ROS homeostasis (Foyer and Noctor, 2003), and are likely to contribute to the cellular self-protection against drought-inflicted damages through detoxification of ROS. Thus, peroxisome proliferation is a mechanism that is involved in oxidative ROS (Palma et al., 1991). Meanwhile, Castillo et al. (2008) observed an interaction between JA and peroxisome proliferation in response to wounding in Arabidopsis, and the impact of JA on the size of peroxisomes was documented in their study. The enlarged peroxisomes that were observed in our work is another known phenotype of peroxisome proliferation, which has been documented previously (Mano et al., 2006, 2011). To date, it was reported that *A. thaliana;* a defect in the *PEX11* proteins (Lingard and Trelease, 2006; Koch et al., 2010), or the *DRP3A, DRP3B, and DRP5B* proteins have caused a small number of elongated peroxisomes (Mano et al., 2011). Also, in the mammalian cell, dynamin-like protein *DLP1* has an independent function for peroxisomal elongation, constriction, and fission (Koch et al., 2004). However, the phenotype of enlarged peroxisomes under our drought conditions might be due to the root-to-shoot hormonal signals of JA and the accumulation of ABA, or an up-regulation of some important genes that are involved in the peroxisome elongation phase.

The timing and scale of the plant response to drought are dictated by stress severity and duration (Shinozaki et al., 2015). The tolerant genotype (Onas) may have a unique mechanism. It can have maintained a balance between the water loss and water potential regardless of the absence or presence of slight stomata closure, or even limited ROS accumulation at the early drought phases. In addition to its high capacity for sensing stress signals and proliferate peroxisomes for an early adaptive response as an early adaptive mechanism. In comparison to the late response in the susceptible genotype (Alpowa). During an advanced phase of drought, Alpowa had an elevated peroxisome proliferation, dramatic stomata closure, and ROS accumulation, which is associated with plant death symptoms. Clearly, limited ability for maintaining an early defense mechanism was shown in the presence of programmed cell death (PCD).

Generally speaking, PEX11s genes are known to promote peroxisome division machinery in association with DRP3A, DRP3B, and DRP5B, and FIS1A (Yan et al., 2005; Aung et al., 2010). Abiotic stresses stimulate the expression of PEX11s genes and DRP5B (Willekens et al., 1997; Nayidu et al., 2008; Fahy et al., 2017). As a novelty in the present study, the gene expression of mentioned genes was assessed during drought phases in the adaptive and non-adaptive genotypes. DRP3A genes correlated with the stomatal opening in Onas. While DRP5B gene has shown a correlation with the stomatal closure in Alpowa. In a recent study, it has been found that DRP5B (ARC5) gene affects the plastid replication (Fujiwara et al., 2018). The up-regulated expression of all studied genes except DRP3A and DRP3B genes under severe drought (during phase III) emphasizes a significant role in the adaptive performance of Onas, with a higher focus on PEX11-4 and−5 and DRP5B genes.

Here, the summary of the main evidences that peroxisome abundance can be considered as a potential cellular trait: (1) the negative correlation with grain yield and other phenological parameters, (2) the high sensitivity based on the ROC analysis, (3) the high value of broad-sense heritability in non-stressed conditions (84.49%), and (4) the increase in heritability under stressed conditions to (88.47%), (5) the upregulation of peroxisome proliferation machinery genes was associated with drought-adaption.

Generally, this study contributes to expanding knowledge on understanding the differential performance of a significant set of phenological traits in response to severe drought. The study enriches the information of drought phenotyping and reduces the overall dimension of the phenological traits for phenotyping drought. It also highlights some parameters that have had less attention for phenotyping drought tolerance and introduces a tool for phenotyping new trait of peroxisome abundance in plants. In particular, the findings in this study reveals that (1) severe drought stress induces peroxisome proliferation and impact the size of peroxisomes, (2) abundant peroxisomes under severe drought stress seems to be genetically programmed, (3) there is negative correlation between peroxisome abundance and yield components, (4) peroxisome abundance can be counted as a cellular trait for phenotyping the plant response under drought, (5) the heritability value of peroxisome abundance is high and increase under drought, thus it can be used as a proxy for genotype selection under drought, (4) there are dynamic changes of the phenological traits during drought phases to distinguish between the adaptive and non-adaptive response, (5) the dynamics of ROS homeostasis seems to be timing dependent mechanism, the adaptive response is earlier than the non-adaptive genotype.

## Author Contributions

MS coordinating the collaboration, conducting the experiments, study investigation, data analysis, creating and writing of original and edited draft. MS, AS, and KG-C conceptualization, methodology, reviewing and editing. KG-C and MS main funding acquisition. KG-C funding the publication fees. AS funding the peroxisome experiments and analysis.

### Conflict of Interest Statement

The authors declare that the research was conducted in the absence of any commercial or financial relationships that could be construed as a potential conflict of interest.
