# Supplementary material for: Differential Dynamic Changes of Reduced Trait Model for Analyzing the Plastic Response to Drought Phases: A Case Study in Spring Wheat
Source: Front Plant Sci. 2019 Apr 26;10:504. doi: 10.3389/fpls.2019.00504 (PMC6497792; doi:10.3389/fpls.2019.00504)
Supplement: Supplementary file 1 [file Table_1.docx]

**Supplementary Tables**

**Supplementary Table 1.** List of genotypes names, samples ID, plant species, release date, origins, wheat type and unique phenotypic features of the used plant materials. The following abbreviations S, HRS, SWS, and DS refer to spring wheat, hard red spring wheat, soft spring wheat, and durum spring wheat, respectively.

| **Genotype** | **ID** | **Plant species** | **Year** | **Origin** | **Wheat**  **type** | **Unique phenotypic** | |
| --- | --- | --- | --- | --- | --- | --- | --- |
| Perigee | NASA | *T. aestivum* cv.  USU-Perigee |  | NASA-USA | S | Dwarf, short coleoptile length, low emergence, short peduncle, length, low biomass, low root dry weight |  |
| Vandal | PI546056 | *T. aestivum* | 1991 | Idaho  AES; USDA-ARS | HRS | Semi-dwarf, |  |
| PWB343 | BW26864 | *T. aestivum* | 1994 | India | S | low tillers number |  |
| Klein dragon | CM64693 | *T. aestivum* | 1992 | Argentina | S | High root dry weight |  |
| PotamS-70 | BW623 | *T. aestivum* |  | CIMMYT | S | High tillers number, low grain number and weight |  |
| Indian | [CItr4489](http://wheat.pw.usda.gov/cgi-bin/GG3/report.cgi?class=germplasm;name=CItr4489) | [*T. aestivum*](http://wheat.pw.usda.gov/cgi-bin/GG3/report.cgi?class=species;name=Triticum+aestivum+ssp.+aestivum) | 1917 | USA-Utah | SWS | Tall plant, short awn length, low grain number and weight, late flowering |  |
| Onas | [CItr6221](http://wheat.pw.usda.gov/cgi-bin/GG3/report.cgi?class=germplasm;name=CItr6221) | *T. aestivum* | 1918 | South Australia | SWS | Awnless, short root and coleoptile length, high tillers number, long peduncle length, high biomass, high root dry weight, high grain number and weight |  |
| Edmore | CItr17748 | *T. turgidum*  var. durum | 1978 | USA-North Dakota | DS | Tall plant, long root length, long coleoptile length, long peduncle length, long awn length |  |
| Sakha93 | Egyptian Cltr | *T. aestivum* | 1993 | Egypt | S | Low biomass |  |
| Sakha94 | Egyptian Cltr | *T. aestivum* | 2004 | Egypt | S | Long root length |  |
| Gemmiza7 | Egyptian Cltr | *T. aestivum* | 2000 | Egypt | S | Low tillers number, low biomass, low root dry weight, low grain number and weight |  |
| Gemmiza9 | Egyptian Cltr | *T. aestivum* | 2000 | Egypt | S | High biomass, high root dry weight, high grain number and weight |  |
| Gemmiza10 | Egyptian Cltr | *T. aestivum* | 2004 | Egypt | S |  |  |
| Sids1 | Egyptian Cltr | *T. aestivum* | 1996 | Egypt | S | Long peduncle length |  |
| Louise | PI 634865 | *T. aestivum* | 2005 | Washington  AES; USDA-ARS | SWS | medium late maturity, semi-dwarf |  |
| Alpowa | PI 566596 | T. aestivum | 1994 | Washington  AES; USDA-ARS | SWS | medium late maturity, semi-dwarf, high tillers number, high biomass, high grain number and weight |  |

**Supplementary Table 2.** information sheet for the predicted peroxisome proliferation machinery genes in *Triticum aestivum* including; gene nomenclature, chromosome position, type of sequence, and the predicted sequence.

| Gene  nomenclature | Chr.  position | Type of sequence | Predicted Sequence |  |
| --- | --- | --- | --- | --- |
| *TaPEX11.3* | Ch. 4 | Transcript | ATGCGGCTGGCGGAGGGTTGTGGGGAGGAGAACCAGGCGGTGACGACGAT  GCGGGGGAAGCTGCTGCTGAAGCGTCTGTCCGTTGTGCAGGACGTGGCGG  ATGCCTTCATGGCGCTAGGGGACGTGACTAACGGGAAGGGGTTGCTCGGT  AGTTCCACGCTGACGGCGTCCGCCGGATTGCTGTCGGCGCTGATCAGCAC  GCACAAGAACTGGAATTCTTGC |  |
|  |  | Protein | MASEARKTAAAARPPPRDFLADLEAYLARRDGVDKLLKISRYAARLALAA  GPLPPPASARLKSFESSLGLSRKAFRLGKFVQDVNALRAHPGPLPPPFVL  LAYGGEGVYYFIEQFVWLAKAGLLPAHLLPRLQCLSAWAELLGYVGSITI  KLEEVTKMESSIKMRRAEGCGEENQAVTTMRGKLLLKRLSVVQDVADAFM  ALGDVTNGKGLLGSSTLTASAGLLSALISTHKNWNSC |  |
| *TaPEX11.4* | Ch. 2 | Transcript | GCCGGCGACACGCTGGACAAGCTGGTGGTGTTCCTGGCGAAGCGCGACGG  CGTCGACAAGCTGGTGAAGACGTACCAGTACGTGGCGAAGCTGGCGCACT  GGGCGGCGGAGACGTCGCACCCGGGGCTGGCGGGGCGCGCCAAGAGCTGG  GAGACGGCGGCCGGGCTGAGCCGCAAGGTGTTCCGGTCGGGCCGGTCACT  GACGGGCTTCAACGCGCTGCGGCGGTCCCCGGGGGAGTTCGGCGCGCTGG  CCGTGCTGGCCAACGCCGGCGAGATGGTCTACTTCTTCTTCGACCACTTC  ACGTGGCTGTCGCGCGTGGGCGTGCTGGAGCCCTGGCTGGCGCGCCGCGC  GAGCTTCGTGTCGGCGTTCGGCGAGTGCGTCGGCTACGTCTTCTTCATCG  CCATGGACTTCATCATGATCAGGCGCGGGATCAGGCGGGAGAGGGCGCTG  CTGCGCGGCGGCGAGGGGAAGGAGGAGGAGAGGGAGGGCGAGGTGCGGAG  GATCCGGGCGGACCGGGTGATGCGGCTCATGGGGACGGCGGCCAACCTGG  CGGACCTGGTCATCGCCGTCGCGGACATCGAGCCCAACCCGTTCTGCAAC  CACGCCGTCACGCTGGGGATCAGCGGCCTCGTCTCCGCCTGGGCTGGGTG  GTATAGGAACTGGCCGTCGTGAG |  |
|  |  | Protein | MSGGGAGDTLDKLVVFLAKRDGVDKLVKTYQYVAKLAHWAAETSHPGLAG  RAKSWETAAGLSRKVFRSGRSLTGFNALRRSPGEFGALAVLANAGEMVYF  FFDHFTWLSRVGVLEPWLARRASFVSAFGECVGYVFFIAMDFIMIRRGIR  RERALLRGGEGKEEEREGEVRRIRADRVMRLMGTAANLADLVIAVADIPN  PFCNHAVTLGISGLVSAWAGWYRNWPS |  |
| *TaPEX11.5* | Ch.7&4 | Transcript | ATGGCCTCATTGGACACCGTCAGAGGAGATCTTGGCCTGGTTGTTTTGTA  CCTAAGCAAGGCTGAGGCAAGAGATAAGATCTGCAGAGCTATACAATATG  GATCCAAGTTCCTGAGCAACGGACAACCAGGACCTGCACAGAATGTCGAC  AAATCAACTAGTCTTGCTCGGAAAGTTTTCCGGCTGTTTAAGTTTGTTAA  TGATCTCCAAGCTCTGATTAGCCCCCCTGCCAAAGGAACTCCACTTCCGC  TGATCTTACTTGGAAAGTCGAAGAACGCGATGCTGTCAACTTTCCTCTTT  CTGGACCAAATTGTCTGGGCTGGGAGAACAGGAGTATACAAGAACAAGGA  GCGAGCAGAATTCCTTGGCAGGATTGCCTTTTATTGCTTTCTTGGATCTA  ATACATGTACTACCATCATCGAGCTAGCGGAGCTTCAGCGGCTTTCCAAA  TCGATGAAGAAGTTAGAGAAGGACCTCAAGCACCAAGAGCTGTACAAGAA  CGAGCAGTATCGGATGAAGCTGAAGAAGTCCAATGAGAGGCTGCTTGCTC  TCATCAAATCGAGCCTTGACATAGTTGTCGCAGTGGGGCTGCTGCAACTG  GCCCCGAAAAAGGTCACTCCTCGCGTCACTGGGGCCTTTGGGTTCGCTAG  CTCGCTCATCGCCTGTTACCAGTTGCTTCCAGCCCCAGCCAAATCCAAG |  |
|  |  | Protein | MASLDTVRGDLGLVVLYLSKAEARDKICRAIQYGSKFLSNGQPGPAQNVD  KSTSLARKVFRLFKFVNDLQALISPPAKGTPLPLILLGKSKNAMLSTFLF  LDQIVWAGRTGVYKNKERAEFLGRIAFYCFLGSNTCTTIIELAELQRLSK  SMKKLEKDLKHQELYKNEQYRMKLKKSNERLLALIKSSLDIVVAVGLLQL  APKKVTPRVTGAFGFASSLIACYQLLPAPAKSK |  |
| *TaFIS1A*  *Or*  *TaBIGYIN* | Ch. 1 | Transcript | ATGGAGGCGCAGATGAGCAAGTTCTTCGAGTCGGTGGGCTCCTTCTTCTC  CGGCGGCGACAACATCCCCTGGTGCGACCGCGACATCATCGCCGGATGTG  AAAGAGAGGTTGCTGAGGCTGCAACCGAAGAACAGAAAAATGATAGCATT  ATGAGGCTATCTTGGGCTCTCGTCCATTCCAAGCAGACTGATGATGTGAA  CCGTGGAATTGGCATGATTGAAGCTTCTCTTGATAAAACCACCAGCCCAC  TGCAGACTAGAGAAAAACTGTATTTGCTGGCTGTTGGGCACTACAGAAAT  GGTAACTATGTAAGGAGCCGGCAACTTGCGGACCGTTGTTTGGAGATTCA  ACCAGATTGGAGGCAGGCATCATCTCTGAAGAAGGCAATAGAGGATAAAA  TTGCTAAAGATGGTGTGATCGGCATAGGAATCGCCACAACTGCGGTAGGA  CTTATCGTTGGTGGGATCGCAGCTGCTCTTGCAAGGAAGAAG |  |
|  |  | Protein | MEAQMSKFFESVGSFFSGGDNIPWCDRDIIAGCEREVAEAATEEQKNDSI  MRLSWALVHSKQTDDVNRGIGMIEASLDKTTSPLQTREKLYLLAVGHYRN  GNYVRSRQLADRCLEIQPDWRQASSLKKAIEDKIAKDGVIGIGIATTAVG  LIVGGIAAALARKK |  |
| *TaDRP3A* | Ch. 2 | Transcript | ATGGCTGACTCGGCAGCGGCTGCTGCTGCGGCGGCGGCGCAACCGTCGAC  AGTGGGGCAAGCCGTGATCCCGCTCGTCAATAGGCTGCAAGACATCATGG  CGCGGCTGGACGGTGACGCCGCCGCCGGCGTGGAGCTGCCACAGGTTGCG  GCGATCGGCGGGCAGAGCAGCGGCAAGTCGAGTGTGCTGGAGGCGCTCGT  CGGCCGCGACTTCCTCCCGAGGGGCCCCGAAATCTGCACGCGCCGCCCCC  TCGTGCTCCAGCTCGTGCGCCACTCGGCCCCCGAGGAGTGGGGCGAGTTC  CTCCACGCCCCCGGCCGCCGATTCGACGATTTCGAACACATCAAGCGCGA  GATCCAGTCGGAAACGGACAAAGAAGCTGGAGGTAACAAAGGTGTCTCTG  ACAAACAGATTCGTCTGAAAATCTTCTCACCAAATGTAATTGACATCACC  TTGGTTGACCTCCCTGGAATTACAAGGGTTCCGGTCGGAGATCAGCCTAG  TGATATTGAGTCAAGAATAAGAACAATGATCATGCAATACATCAAGCATC  CAAGCTGCATTATCTTGGCCGTCTCACCCGCAAATGCAGATTTAGCCAAT  TCTGATGCTCTTCAACTGGCACGGCTTGGTGATCCTGATGGATCTCGTAC  AATTGGTGTTATCACCAAGTTGGACATCATGGACAGGGGTACTGATGCTC  GTAACTTTTTACTGGGAAATGCAATCCCCCTCAAGCTTGGTTATGTAGGT  ATTGTGAATCGCAGCCAAGAGGACATCAACTTTAACCGAAGCATCAAAGA  TGCACTTGCCTTTGAGGAGAAGTTTTTCTCGACTCTACCTGCTTATCATG  GTCTTTCACAATGTTGTGGTGTTCCTCAATTGGCCAAGAAGTTAAATATG  ATTCTACTAAAGCACATCACAGATATGCTTCCAGGTTTGAAATCTCGAAT  AAATGCTCAGTTGGTAGCAGTTGCCAAGGAACATGCTGCATATGGTGATA  CGGCAGAATCGACGGCTGGTCAGGGAGTCAAACTATTGAACATATTGGGA  AAATATTGTGAAGCTTTTTCTTCGATGGTGGAGGGAAAAAATAAAGTGTC  AACAGATCAGCTTTCTGGTGGAGCAAGAATTCACTACATTTTTCAGTCAA  TTTTTGTCAAAAGCTTGGAGGAAATTGACCCTTGCAAGAATATAAGCGAT  GAAGATATCCGCACGAGTATACAGAACTCTGACGGTCCAAAGGGTGCTAT  GTTTCTGCCAGAGGTGCCTTTTGAGATTCTTGTGCGAAAGCAGATAGGCC  GTTTGCTTGATCCAAGTCTTCAGTGTGCCAAATTTATCTATGACGAGTTA  GTCAAAATTAGCCATGGTTGCTTAACTAGTGAGCTGCAGAAATACCCAAT  TCTTAAAAGACGGATGGGTGAATCAGTTAGCAATTTCTTGAGAGATGGTC  TTCGACCTGCGGAGACAATGATAACTCATATTATTGAAATGGAGATGGAT  TACATAAATACCTCACATTCAAGCTTTATTGGAGGCAGCAAGGTTGTTGA  ACTTGCTAAGCACGAAGGTCTATCTTCGAGAGGACCAACTTCACTATCGG  TTCATAAGGATGGTGTTGGTATAAGTTCTGAGGCGCAGCTAAAATCTTCT  ACTGAGAACAATGTACAGCTCAAATCTGAAAGAGGTCAAAAGTCACGTGC  TGTTTTTGCAAGAGATACTTCCAGAGGAGCAACAGCTGAGAAGGGATTTC  AGCCTGATACAGATGCAGGAACAAGTGTGGCAGGTGGAGGCCAGAATGGT  CACTCACTTGTTAGTGGGAGTTTGTCAAGCATGTCAGATCCACGAGGTTA  CAGCCTCAATAGCTTATACTCTATGATTCGGTTAAGAGAGCCACCGAGCA  CCTTGAAACCATCAGAAAACAAGACTGACCGGGACAGAACAGAGATAGCT  ATTGTGAAGCTTTTGGTCAAATCTTACTATGACATTGTCAGAAAGAGTAT  TGAGGATGCGGTTCCCAAAGCTATAATGCATTTTTTGGTGAACCACACAA  AGCGGGAGCTCCACAACGTTCTAATTCGGAAACTGTACAGGGAGAGCCTA  CTTGATGACATGCTGAGGGAAACAGATGAAGTACTTATCAGACGGCAGCG  TATTCAAGAAACGCTCCAAGTTCTTGAACAGGCACACAGGACGCTTGAGG  AATTTCCCCTTGAAGCTGAGAAGCTTGAGAGGGGCTACAGCCTCTCTGAG  TATGGCACTGGCCTGCCGAATATCCCTGGACTCAGCAATCGCAATCCTAG  GGGTATCCTCCCT |  |
|  |  | Protein | MADSAAAAAAAAAQPSTVGQAVIPLVNRLQDIMARLDGDAAAGVELPQVA  AIGGQSSGKSSVLEALVGRDFLPRGPEICTRRPLVLQLVRHSAPEEWGEF  LHAPGRRFDDFEHIKREIQSETDKEAGGNKGVSDKQIRLKIFSPNVIDIT  LVDLPGITRVPVGDQPSDIESRIRTMIMQYIKHPSCIILAVSPANADLAN  SDALQLARLGDPDGSRTIGVITKLDIMDRGTDARNFLLGNAIPLKLGYVG  IVNRSQEDINFNRSIKDALAFEEKFFSTLPAYHGLSQCCGVPQLAKKLNM  ILLKHITDMLPGLKSRINAQLVAVAKEHAAYGDTAESTAGQGVKLLNILG  KYCEAFSSMVEGKNKVSTDQLSGGARIHYIFQSIFVKSLEEIDPCKNISD  EDIRTSIQNSDGPKGAMFLPEVPFEILVRKQIGRLLDPSLQCAKFIYDEL  VKISHGCLTSELQKYPILKRRMGESVSNFLRDGLRPAETMITHIIEMEMD  YINTSHSSFIGGSKVVELAKHEGLSSRGPTSLSVHKDGVGISSEAQLKSS  TENNVQLKSERGQKSRAVFARDTSRGATAEKGFQPDTDAGTSVAGGGQNG  HSLVSGSLSSMSDPRGYSLNSLYSMIRLREPPSTLKPSENKTDRDRTEIA  IVKLLVKSYYDIVRKSIEDAVPKAIMHFLVNHTKRELHNVLIRKLYRESL  LDDMLRETDEVLIRRQRIQETLQVLEQAHRTLEEFPLEAEKLERGYSLSE  YGTGLPNIPGLSNRNPRGILP |  |
| *TaDRP3B* | Ch.3&5 | Transcript | ATGGCCGACGACCACTACAGCTCCGCCGCCTCCGCGCCGTCGCCGGCGGC  GGCGGCGGCGGCCGAGGCCGCCGCGGTGGTGGGCTCCTCGGTGATCCCGA  TCGTCAACAAGCTGCAGGACATCTTCTCCAGCTGGGGAGCAGCTCCACCA  TCGACCTGCCGCAGGTGGCCGTCGTCGGCAGCCAGAGCAGCGCAAGTCGA  GCGTCCTCGAGGCGCTCGTCGGCCGCGACTTCCTCCCGCGCGGCTCCGAC  ATCTGCACGCGCCGCCCGCTCGTGCTCCAGCTCGTGCACCAGCCGCGCCG  CCCCGCCGACGCCGAGAGGGACGAGTGGGGCGAGTTCCTGCACCACCCTG  GCCGCCGCTTCTACAAGTTCCCCGACATCCGACGCGAGATCCAGGCAGAA  ACGGACAGGGAGGCTGGCGGTAACAAGGGCGTCTCAGATAGACAGATACG  TCTGAAGATCTATTCGCCAAATGTTCTCAACATCACTTTGGTCGATCTGC  CTGGAATTACTAAGGTGCCTGTTGGAGACCAACCAACTGACATTGAGGCC  AGAATAAGGACAATGATATTGTCATATATCAAGCACAAGACATGCATCAT  ATTGGCTGTTTCTCCAGCAAATGCAGATTTAGCGAACTCTGATGCTCTTC  AAATGGCTCGGCAAGCTGATCCTGATGGTTCTCGAACAATTGGTGTTATC  ACGAAGTTGGACATAATGGACAGGGGCACTGATGCCCGTAATTTTTTGCT  GGGGAATGTCATCCCATTACGACTTGGTTATGTTGGCGTCGTAAACCGCA  GCCAGCAGGATATCAATTCAGATGTCAGTGTCAAGCAGGCTCTGGCCCGA  GAAGAAAGTTTCTTCCGTACTCATCCGGCATACAATGGTCTTGCTAAGCA  CTGTGGGATACCACAGTTAGCAAAGAAGCTAAACCAGATCTTGGTCCAAC  ATATAAGAACTATTCTGCCTGGACTGAAGGCACGCATAAGTTCTCAATTG  ACAGCTATTGCTAAGGAGCATGCCTTTTATGGTGACCCAGTCGAGTCCAA  GGCTGGGCAAGGTGCTAAGCTTTTGAACATTCTAGCTAAATACTGTGATG  CTTTCTCGTCTATGGTAGAAGGAAAAAACGAAGATATATCAACAATCGAG  CTTTCTGGTGGAGCAAGAATTCACTATATTTTTCAATCTATCTTTGTCAA  AAGCTTAGAGGGTGTTGACCCTTGCGAGGATGTCACTGATGAAGATATCC  GCATGGCTATACAAAATGCAACTGGTCCTAGGAGTGCTTTGTTTGTACCT  GAGGTGCCATTTGAAGTCCTTGTACGCAGGCAAATCAGCAGATTGCTTGA  TCCAAGTCTTCAGTGTGCAGATTTCATATATGAGGAACTGGTTAAGATGA  GCCACCGTTGCCTTTGCAATGAGCTGCAGCAGTTTCCTATACTCAGAAGG  AGCATGGATGAAGTAATTGGGAAGTTTCTACGGGATGGGCTTAAGCCAGC  ACAAGATATGATAGCGCACATCATTGAGATGGAGGCCGATTACATAAACA  CATCTCACCCAAGTTTCATCGGTGGCAGCAAGGCTGTAGAACAAGCACAG  CAACAAGTTAGAGCCGCTAGATTGCCTGCAACAGTGGTTAGAAGGGATGG  AGTAGATGCAGATAGGCCACAGGCTTCTGAAAAAACCCAAAAAGCACGTG  CATTATTGGGTAGAACTACTGGTGTAAATGGAGTTATCACCGACCAGATC  CAGGGGGTACGATCTGCTGCTGAGGCAGAGAGACCAGGATCTTCAGGTAG  TGGAAGCACTTCTTTTTGGGGCTCAATATTCACCTCAAGCGAAGACCGTG  CACATTCTTCAGCAAGAGGTAGCTCAACGAACAAATCTTATGCTTCAGCT  ACCCCCAACCTGGAACATTCATTCTCTTCAATACAGTTAAAAGAGCCACC  GCTAGTCCTCAAGCCTTCAGAAAGTCATTCTGAGCAGGAGGACCTTGAAA  TAGCAATCACAAAATTGCTGCTGCAGTCATACTACAATATAGTCAGGAAA  AATGTTGAGGATTTCGTACCCAAAGCAATTATGCATTTTCTGGTTAATCA  CACGAAAAGGGAGCTGCATAATTTCCTTATAACTACCCTTTATAGAGAAG  AGCTCTTTGGGGACATTCTTAGAGAACCTGACGAAATAACTACAAAGAGG  AGGCAGATACGCGATACTCTTAAGGTCCTTCAGCAAGCCTACAAAACTTT  GGACGAGATACCGCTTGAAGCGGAGACGGTCGAGAGGGGCTATTCCCTGG  ATTCTGATGCGACGGGTCTACCACGGGTCCATGGGGTTTACGATGGAAGC  TCACCATATTCGACTCCGAAGCAAACGAGGCCTAGGAAATCAAGCCACTC  CGGGGAGCAGCAACAACCTTTCAGTGGCAATGGGTTT | |
|  |  | Protein | MADDHYSSAASAPSPAAAAAAEAAAVVGSSVIPIVNKLQDIFSQLGSSST  IDLPQVAVVGSQSSGKSSVLEALVGRDFLPRGSDICTRRPLVLQLVHQPR  RPADAERDEWGEFLHHPGRRFYKFPDIRREIQAETDREAGGNKGVSDRQI  RLKIYSPNVLNITLVDLPGITKVPVGDQPTDIEARIRTMILSYIKHKTCI  ILAVSPANADLANSDALQMARQADPDGSRTIGVITKLDIMDRGTDARNFL  LGNVIPLRLGYVGVVNRSQQDINSDVSVKQALAREESFFRTHPAYNGLAK  HCGIPQLAKKLNQILVQHIRTILPGLKARISSQLTAIAKEHAFYGDPVES  KAGQGAKLLNILAKYCDAFSSMVEGKNEDISTIELSGGARIHYIFQSIFV  KSLEGVDPCEDVTDEDIRMAIQNATGPRSALFVPEVPFEVLVRRQISRLL  DPSLQCADFIYEELVKMSHRCLCNELQQFPILRRSMDEVIGKFLRDGLKP  AQDMIAHIIEMEADYINTSHPSFIGGSKAVEQAQQQVRAARLPATVVRRD  GVDADRPQASEKTQKARALLGRTTGVNGVITDQIQGVRSAAEAERPGSSG  SGSTSFWGSIFTSSEDRAHSSARGSSTNKSYASATPNLEHSFSSIQLKEP  PLVLKPSESHSEQEDLEIAITKLLLQSYYNIVRKNVEDFVPKAIMHFLVN  HTKRELHNFLITTLYREELFGDILREPDEITTKRRQIRDTLKVLQQAYKT  LDEIPLEAETVERGYSLDSDATGLPRVHGVYDGSSPYSTPKQTRPRKSSH  SGEQQQPFSGNGF | |
| *TaDRP5B*  *OR TaARC5* | Ch. 5 | Transcript | GAAGGAAATTATAATAAGGATAGAATATAAACATTGCCCCAATCTCACCA  TTATTGACACTCCAGGTCTCATTCTTGCAGCTCCTGGCCGTAAAAACCGA  GTTTTGCAGAGTCAGGCTTGTGCTGTTGAGGCCCTTGTTCATGCGAAAAT  CCATCATAAAGAACCATAATTCTGTGCCTTGAAGATTGTAGTGATTGGAG  CAATGCAACTACAAGGAGAGTGGTGATGCAGGTTGATCCTGATCTTGCAA  GGACTGTTCTAGTCTCCACAAAACTTGACACGAAAATTCCACAATTCGCA  CGTGCCTCTGATGTTGAAGTGTTTCTTCACCCACCAACTTGTGTTCTAGA  TGGTTCCTTATTGGGAGATTTTTCCCTTTTTCACATCAGTGCCTTCTGGG  AGAGTTGGTTCTTGCCATGAAGCTGTCTTCAGATCAAATGAAGAGTTTAG  AAGGCGATCTCACTGAGAGAATTGGATGATGTTACATCTCTTGAAGACAA  GCTAGGAAGGTCACTTACAAGGGAGGAGAAGAACAGGATAGGAGTGAGTA  ACTTAAGATTATTTTTGGAAGAATTGCTGCAGAACAGGTACATCGAGAGT  GTTCCGTCGATTATTCCACTTCTTGAGAAGGAACATCGCGCTGCATCGAG  GAAGTTGCGCAAAGTCACACAAGAAATCAGTGATTTGGACGAAGCAAAAC  TAAAGGAGAAAGCTCGCCTGTTCCATGATTCATTTTTGACAAAGTTATCT  TTGCTACTGAAAGGCATGGTGGTGGCACCTCCTGATAAGTTTGGAGAAAC  TTCAGTTAACGAGAGGATCAATGGAGGGACATTTACTGGAAGCGAGAATT  TCCAGCTCCCAAACAAGATGATGCCTAATGCTGGAATGCGTCTTTACGGT  GGTGCACAATACCATCGGGCAATGGCTGAATTTCGTCTGGTTGTTGGAAG  TATCAAGTGCCCCCCGATTACTAGGGAGGAAATAGTCAACGCCTGTGGTG  TTGAAGATATTCATGATGGGACGAATTACTCCAGGACTGCTTGTGTAATT  GCTGTGGCAAAAGCACGCGACACATTTGAGCCTTTCCTTCATCAGTTGAG  TTTTAGGCTGTTGTACATACTGAAAAGATTGAGTCCGATATCTGTTTTTC  TGCTGGAGAAGGACGGTGAGCACTTTAGCAGCCATGATGTACTTGTGAAG  CGTGTCCAAGCTGCTTTCAACAGGTTTGCTGAATCTACCGAGCAATCTTG  CCGTGAAAGATGTATGGAAGATTTGGAGAGCACCACTCGTTATGTTACTT  GGTCCCTTCACAACAAGAACCGTGCCGGGTTGCGACATTTTCTGGATTCG  TTTGTTGCACCGGAGCAATTATGTGCTAACACACATACTGCCCACTCAGC  TGGGCTGCATGAGCAATCCACTGGTTTGAATGACAGTAAGCAGGATAGAC  CAAAGGGAGAGCTGAAATCTAGTCATACTTCAGACTCAAATCCATCCGGT  GGTGTGTCAGAAACGAGACTGGTGGATCTCTTGGACAATACGCTGTGGAA  CCGAAGGCTGGCGCCCTCATCTGAGCGTCTTGTGTATGCACTGGTTCACC  AGATTTTTCATGGGATCAAAGAGCATTTTCTTGTCACTACAGAACTAAAG  TTCAATTGCTTCCTCCTGATGCCAATTGTTGACAAATTAGCGGCTCTTCT  ACGAGAAGACCTTGAATCAGCGTTTGAAGACGATCTTGACAGCATCTTCA  GCGTCACCGAGCTGCGCCACTCACTTGGGCAGAAGAAGCGAGAGCTGGAA  ATCGAATTGAAGCAGATGAAGCGTTTAAAAGAGAAATTCACAGAAATAAA  TAAGAAGCTCAATTCTCTCCAGGTTAGACA |  |
|  |  | Protein | PFFTSVPSGRVGSCHEAVFRSNEEFKKAISLRELDDVTSLEDKLGRSLTR  EEKNRIGVSNLRLFLEELLQNRYIESVPSIIPLLEKEHRAASRKLRKVTQ  EISDLDEAKLKEKARLFHDSFLTKLSLLLKGMVVAPPDKFGETSVNERIN  GGTFTGSENFQLPNKMMPNAGMRLYGGAQYHRAMAEFRLVVGSIKCPPIT  REEIVNACGVEDIHDGTNYSRTACVIAVAKARDTFEPFLHQLSFRLLYIL  KRLSPISVFLLEKDGEHFSSHDVLVKRVQAAFNRFAESTEQSCRERCMED  LESTTRYVTWSLHNKNRAGLRHFLDSFVAPEQLCANTHTAHSAGLHEQST  GLNDSKQDRPKGELKSSHTSDSNPSGGVSETRLVDLLDNTLWNRRLAPSS  ERLVYALVHQIFHGIKEHFLVTTELKFNCFLLMPIVDKLAALLREDLESA  FEDDLDSIFSVTELRHSLGQKKRELEIELKQMKRLKEKFTEINKKLNSLQ  VRQ |  |

**Supplementary Table 3.** The designed primer sequences for the qRT-PCR using Primer-BLAST software (F refers to the forward primer, and R refers to the reverse primer.

| Gene nomenclature | Primer sequences (5’- 3’) |
| --- | --- |
| *TaPEX11.3* | F: CGCTAGGGGACGTGACTAA  R: CAGCGCCGACAGCAATC |
| *TaPEX11.4* | F: CAACCCGTTCTGCAACCAC  R: TTCCTATACCACCCAGCCCA |
| *TaPEX11.5* | F: GAAGAACGCGATGCTGTCAA  R: TAAAAGGCAATCCTGCCAAG |
| *TaFIS1A* | F: TCCAAGCAGACTGATGATGTG  R: TGGGCTGGTGGTTTTATCAAGA |
| *TaDRP3A* | F: GACCTGCGGAGACAATGATAAC  R: GTTGGTCCTCTCGAAGATAGA |
| *TaDRP3B* | F: TGGACGAGATACCGCTTGAA  R: CACTGAAAGGTTGTTGCTGC |
| *TaDRP5B* | F: AGGGAGGAAATAGTCAACGCC  R: TCAAATGTGTCGCGTGCTTT |
| *TaActinII* | F: CAAATCATGTTTGAGACCTTCAATG  R: ACCAGAATCCAACACGATACCTG |

**Supplementary Table 4.** Pearson correlation among all the measured phenological traits, the algorithms of the robust method are used for calculations.

See the attached excel table
