# Supplementary material for: Differential Dynamic Changes of Reduced Trait Model for Analyzing the Plastic Response to Drought Phases: A Case Study in Spring Wheat
Source: Front Plant Sci. 2019 Apr 26;10:504. doi: 10.3389/fpls.2019.00504 (PMC6497792; doi:10.3389/fpls.2019.00504)
Supplement: Supplementary file 4 [file Data_Sheet_1.PDF]

Supplementary Figures

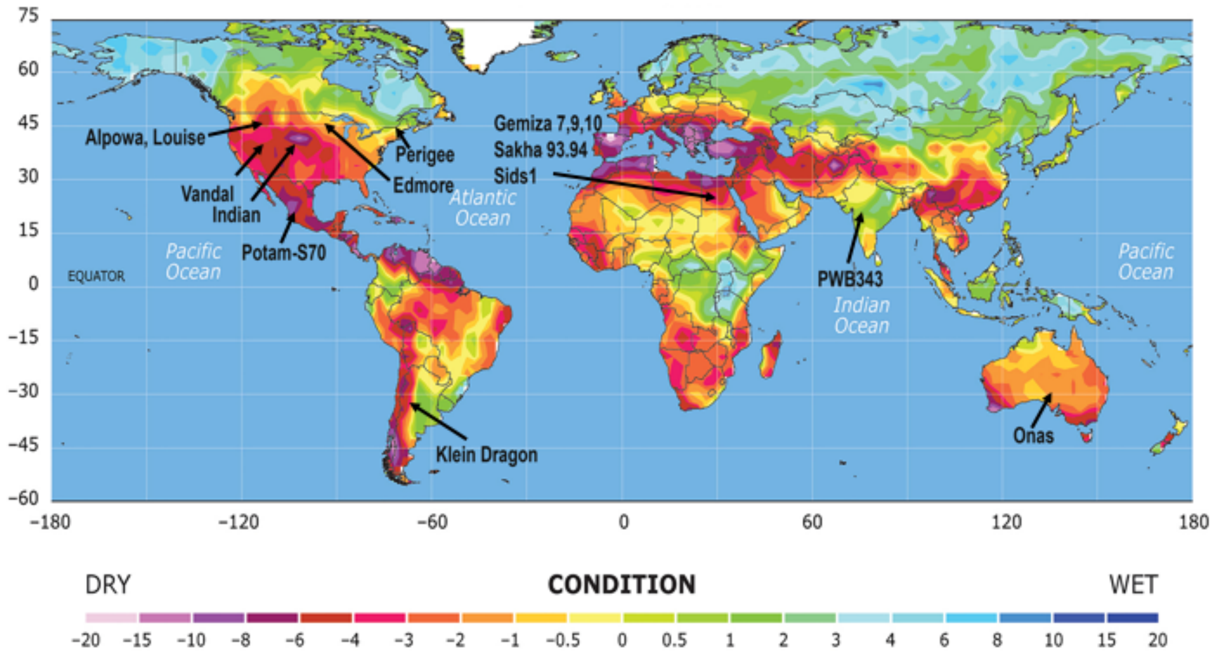

Figure S1. The National Center for Atmospheric Research forecasting of the global distribution of aridity between 2030 to 2039. Severe drought will overrun most of the Americas, Australia, Africa, Southern Europe, Southeast Asia, and the Middle East, based on the Palmer drought severity index (PDSI). Regions were scaled by -4 or below will counter severe drought, and regions that colored by blue or green colors are predicted to be at lower risk of drought. The genotypes were located on the map.

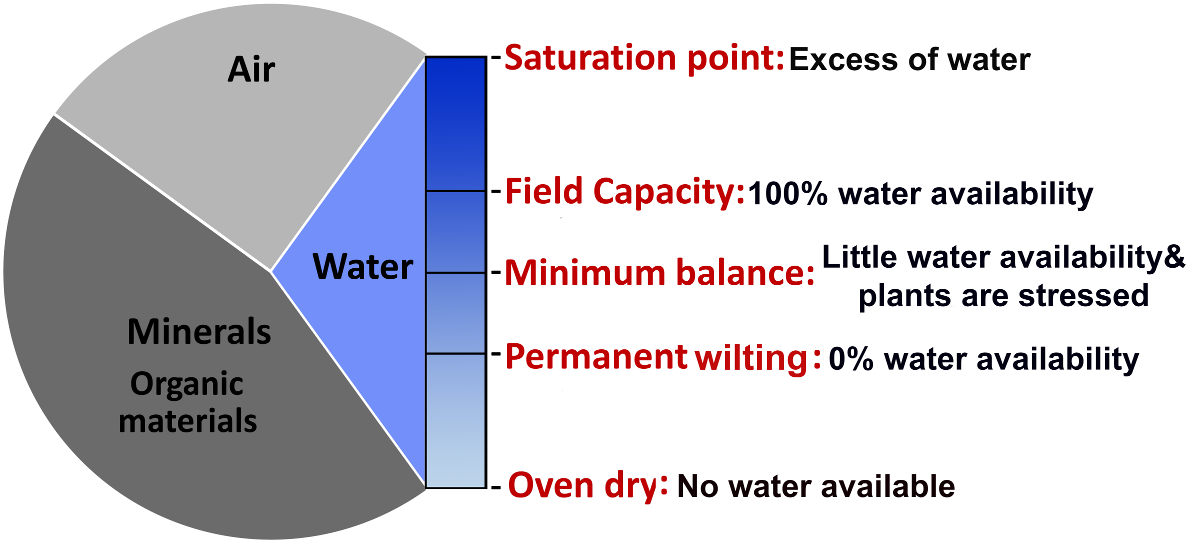

Figure S2. The created figure to identify the soil moisture distribution associated with the readable description of the plant state under different degrees of soil moisture according to the description that mentioned in Werner 2000.

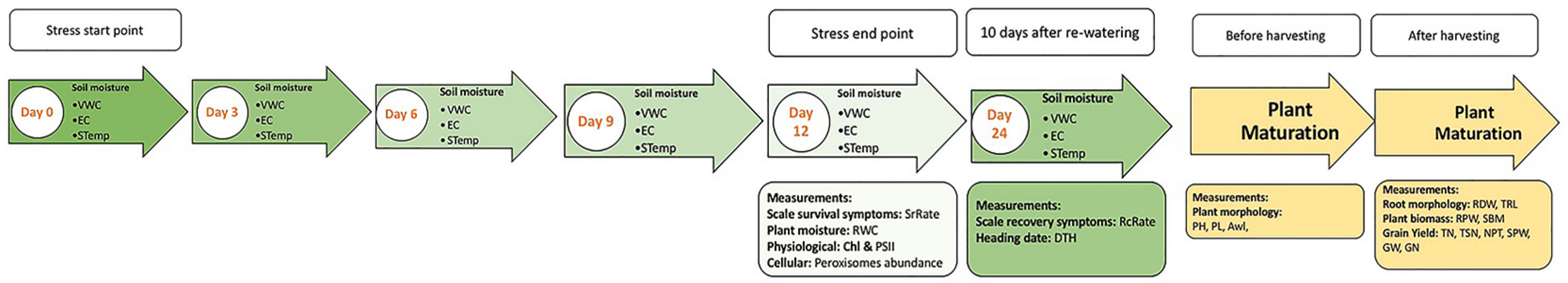

Figure S3. The experiment workflow. After withholding the watering, the soil moisture was measured every three days. The soil moisture measurements are volumetric water content (VWC), water content (RWC), chlorophyll content (Chl), electrical conductivity (EC), and soil temperature (STemp). To phenotype the plants after a prolonged drought, relative leaf temperature (LTemp), photosystem II activity (PSII) and peroxisomes were measured. Twelve traits were estimated after plant maturation to recovery and yield. Shoot dry weigh, plant height (PH), tiller number (TN), peduncle length (PL), awn length (AWL), root dry weight (RDW), total root length (TRL), standing biomass (SBM), total spike number (TSPN), number of productive tillers (NPT), total spike weight (SPW), total grain number (GN), and total grain weight (GW) were estimated.

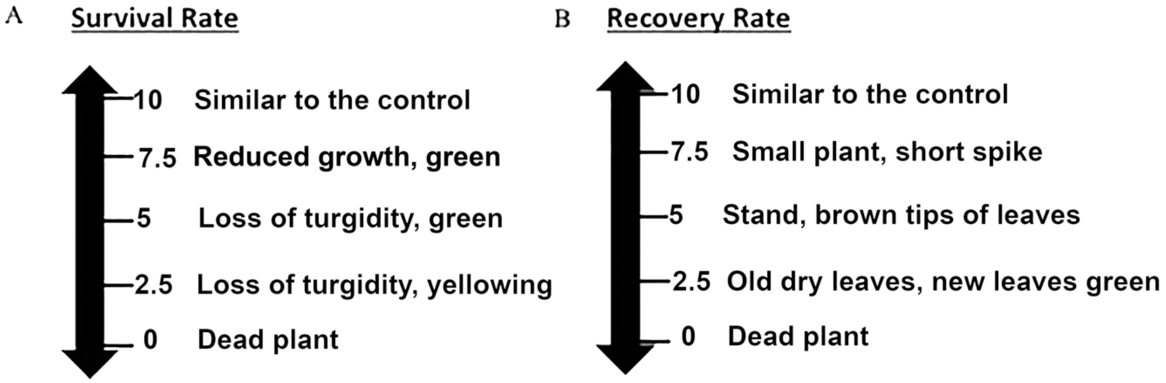

Figure S4. The scale of stress symptoms under severe drought. **(A)** At survival rate, the observations were taken at the maximum stress point (below the permanent wilting point). **(B)** The recovery rate of the plants was evaluated after 10 days of re-watering.

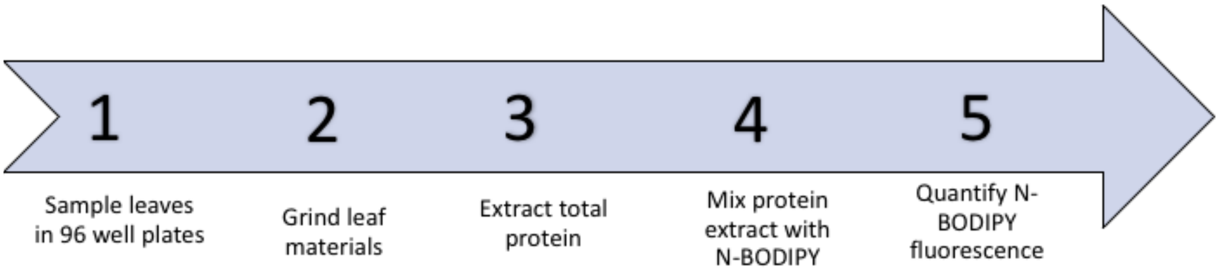

Figure S5. Workflow for measuring the peroxisome abundance in a high-throughput format. The process includes five steps: (1) Collect leaf base in 96 well plates placed in liquid nitrogen. (2) Grind leaf materials in tissue grinder using freezing conditions. (3) Add protein extraction buffer, homogenize, centrifuge, and collect supernatant. (4) Mix the protein extract with the N-BODIPY. (5) Measure fluorescence with a spectrofluorometric plate reader (490 nm excitation, 530 nm emission).

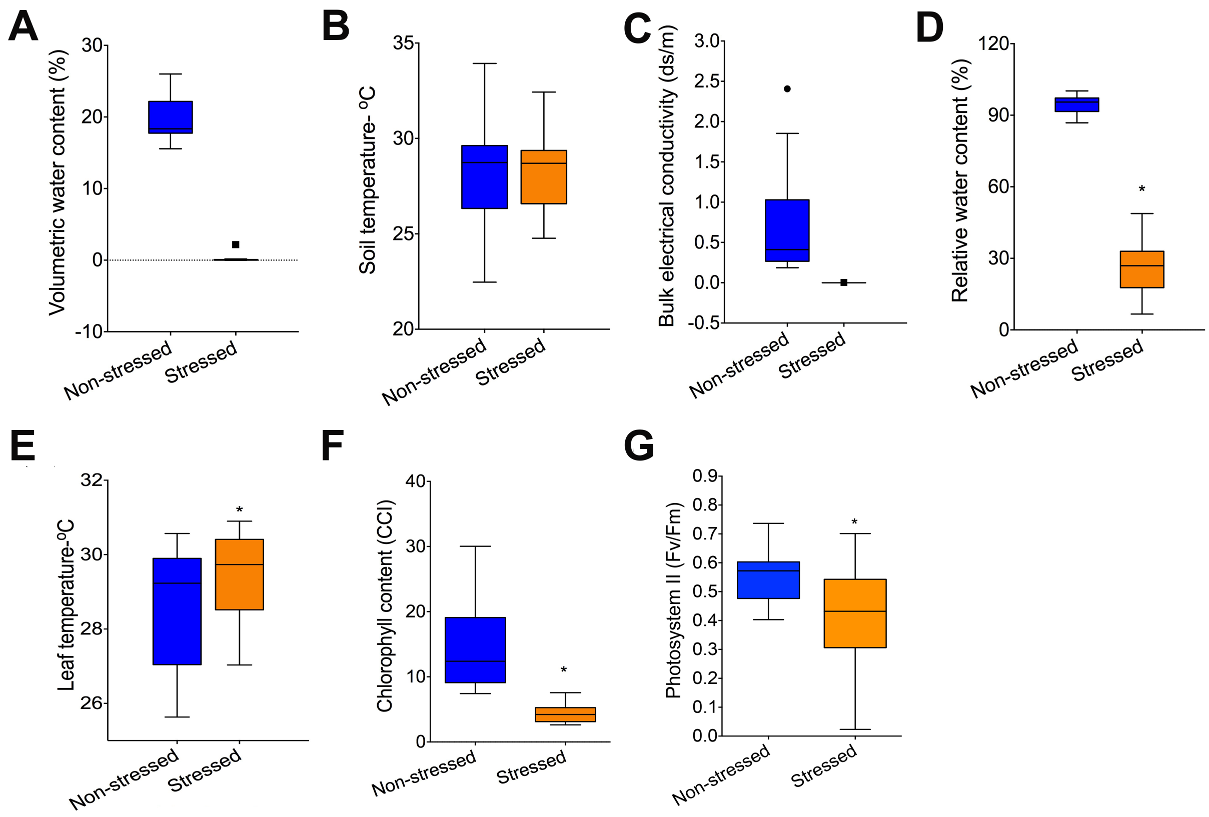

Figure S6. The overall mean of some phenotypic traits on the 12th day after withholding watering. Volumetric water content reached the minimum level **(A)**, in soil temperature **(B)**, soil electrical conductivity **(C)**, the relative water content **(D)**, the leaf temperature **(E)**, the chlorophyll content **(F)**, and the photosynthetic activity **(G)**. Bars represent means ± SD of all the genotypes in each group of treatments, and asterisks display the significant difference at 95% confidence using Tukey's means comparisons test.

Movie

Please find the attached video

Figure S7. A movie to illustrate the streaming of peroxisome abundance in wheat leaves under the normal conditions.

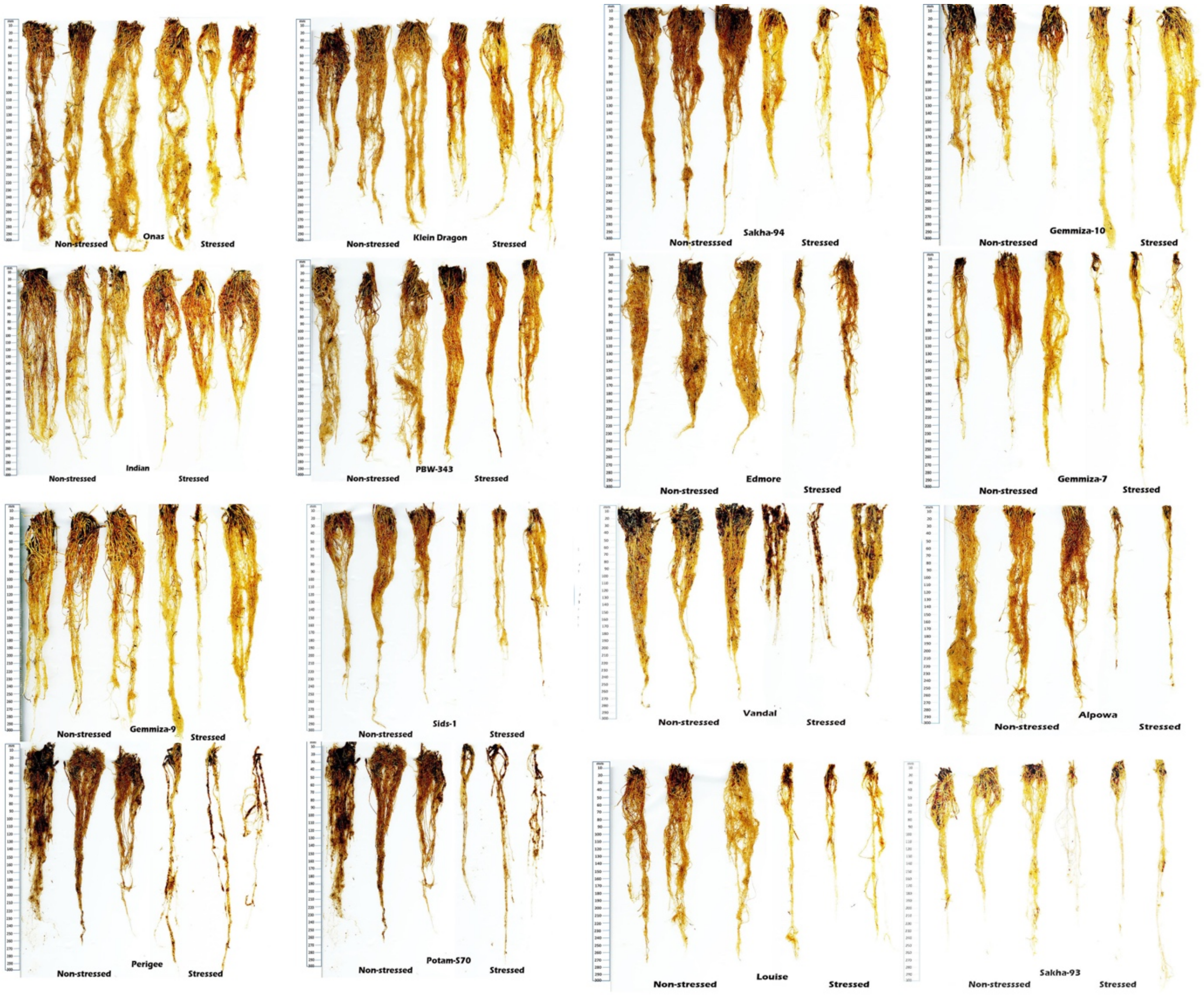

Figure S8. Represented images of non-stressed and stressed roots (3 biological replicates per genotypes and treatment are represented) taken at plant maturity. These images were applied to calculate the total root length using Assess 2.0 image analysis software.

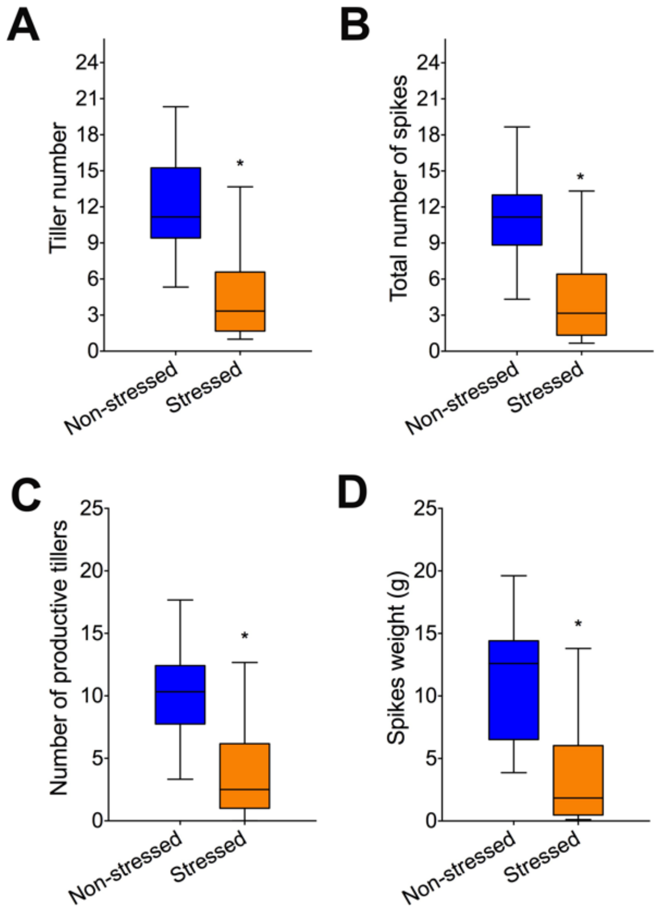

Figure S9. Impact of drought on the yield parameters. **(A)** Tiller number, **(B)** the total number of spikes, **(C)** number of productive tillers, and **(D)** total spike weight. Bars represent means ± SD of all the genotypes in each group of treatments, and asterisks display the significant difference at 95% confidence using Tukey's means comparisons test.
